# Consumer perception of “artificial meat” in the educated young and urban population of Africa

**DOI:** 10.3389/fnut.2023.1127655

**Published:** 2023-04-14

**Authors:** Moïse Kombolo Ngah, Sghaier Chriki, Marie-Pierre Ellies-Oury, Jingjing Liu, Jean-François Hocquette

**Affiliations:** ^1^INRAE, Clermont Auvergne, Université Clermont Auvergne, VetAgro Sup, UMR1213, Recherches sur les Herbivores, Saint Genès Champanelle, France; ^2^Isara, Lyon, France; ^3^Bordeaux Sciences Agro, Feed & Food Department, Gradignan, France

**Keywords:** consumer survey, cultured meat, cell-based food, food security, willingness to engage, livestock

## Abstract

African’s population is expected to grow especially in cities to reach about 2.5 billion in 2050. This will create an unprecedented boom in the demand for animal products over the coming years which will need to be managed properly. Industry stakeholders worldwide have been touting the potential benefits of “artificial meat” in recent years as a more sustainable way of producing animal protein. “Artificial meat” is therefore moving into the global spotlight and this study aimed to investigate how African meat consumers of the coming generations perceive it, i.e., the urban, more educated and younger consumers. Three surveys were conducted with more than 12,000 respondents in total. The respondents came from 12 different countries (Cameroon, Congo, -DRC Democratic Republic of Congo, Ghana, Ivory Coast, Kenya, Morocco, Nigeria, Senegal South Africa, Tanzania and Tunisia). Respondents in this survey prefered the term “artificial meat”. This term was therefore used throughout the survey. “Artificial meat” proved to be fairly well known in the surveyed countries as about 64% the respondents had already heard of “artificial meat.” Only 8.9% were definitely willing to try “artificial meat” (score of 5 on a scale of 1–5) mostly males between 31 and 50 years of age. Furthermore, 31.2% strongly agreed that “artificial meat” will have a negative impact on the rural life (score of 5 on a scale of 1–5) and 32.9% were not prepared to accept “artificial meat” as a viable alternative in the future but were still prepared to eat meat alternatives. Of all the results, we observed significant differences in responses between respondents’ countries of origin, age and education level with interactions between these factors for willingness to try. For instance, the richest and most educated countries that were surveyed tended to be more willing to try “artificial meat.” A similar pattern was observed for willingness to pay, except that gender had no significant effect and age had only a small effect. One major observation is that a large majority of respondents are not willing to pay more for “artificial meat” than for meat from livestock.

## Introduction

The African population is expected to reach about 2.5 billion people in 2050. About 80% of this increase will occur in cities, with nearly 1.5 billion Africans living in urban areas (1). This increase in urban population along with increased income is set to increase the demand for animal-sourced food (2–4).

The livestock sector in some African countries is the fastest growing agricultural subsector (5). It contributes not only to food and nutrition security but also to economic growth by providing important foreign exchange through increased trade within and between African countries, as well as with other regions, such as the Middle East (6). Africa’s livestock accounts for one third of the world’s livestock population (3) and about 40% of agricultural GDP in Africa, ranging from 10 to 80% depending on the country (6).

Unfortunately, African livestock farming systems are less efficient and productive than their counterparts in more developed countries with smallholders being the main suppliers of animal-derived food (6). These smallholders are also far from markets and depend on abattoirs with limited infrastructures, thereby making it difficult to meet the growing demand for meat (7).

Between 2018 and 2020, one African person consumed on average 13 kg of meat per year, with chicken being the most consumed at 5.75 kg *per capita*. By 2030, Africa’s meat consumption is expected to increase by 30% and growth in consumption will outpace the expansion of domestic production (7), and the amount of meat consumed is expected to increase to 26 kg of meat in 2050 (6, 8). Consumption patterns across Africa vary significantly; some countries consume as little as 10 kg of meat per person, around half of the continental average. Countries with higher incomes such as South Africa, consume between 60–70 kg of meat per person (9).

In addition to this demographic pressure, livestock farming systems across Africa will have to cope with climate change and develop sustainable methods of production. African livestock production already has a significant impact on the environment (10). More than 70% of agricultural greenhouse gas emissions in Africa comes from the livestock sector, dominated by enteric methane (CH_4_) emissions (3).

Furthermore, the most significant environmental impacts and nutritional issues associated with animal-sourced food consumption are predicted to occur in Africa and as well as in other low- and middle-income countries of the World (11). It will therefore be a challenge for Africa to produce meat quantitatively and qualitatively for its population. Consequently, there is a huge opportunity for the private sector due to the continent’s swelling dietary needs. There will be a need for the private sector to invest in veterinary services, drugs, vaccines, animal feed and infrastructures. Smallholders, with limited production resources, cannot reach these objectives.

Given this context, and especially with regard to the expected environmental challenge and the growing demand for animal protein in Africa, “artificial meat” appears to be a viable solution as suggested by its proponents. This novel food product makes use of ground-breaking technologies such as tissue culture and bioreactor engineering to increase the production of meat alternatives that may become a threat to the conventional meat industry (12, 13). “Artificial meat” is produced by *in vitro* tissue or cell culture, or by three dimensional (3D) printing of meat (14). “Artificial meat” currently faces its own problems, such as technological barriers, sensory, nutritional, health and safety challenges, in order to be fully accessible to developing food meat markets (15, 16).

“Artificial meat” is a technical revolution, but it could also be considered as a potential economic and societal revolution, which could disrupt the traditional meat sector (17). Consumer acceptance of this novel product has been studied in many European (18–22), American (23, 24) and Asian countries (25), but little is known about the potential acceptance of “artificial meat” in African countries, despite the world’s greatest challenges in terms of meat demand, climate issues and socio-economic challenges as described above.

The aim of this study is therefore to explore the responses of African consumers of the next generations (i.e., mainly urban, more educated and younger consumers) to relevant questions in order to investigate their attitudes, outlook, potential acceptance, and willingness to engage with “artificial meat,” and to provide insight into the factors that may lead to the acceptance of “artificial meat” in the general context of Africa.

## Materials and methods

### Design of the questionnaire

Three surveys were conducted. All surveys adhered entirely to the ESOMAR (European Society for Opinion and Market Research) guideline for ethical online research (ESOMAR, 2011). Indeed, all respondents had to give their explicit informed consent to take part in the survey and their personal data was protected. In addition, respondents’ data was collected in an anonymous way with a “do not wish to answer” option and with no personally identifiable information. This research was conducted in accordance with the published guidelines of the countries in which it was performed with, when required, the approval of ethics committees (such as in Brazil: CAAE number: 37924620.5.0000.5404 (23)).

**Figure 1:**
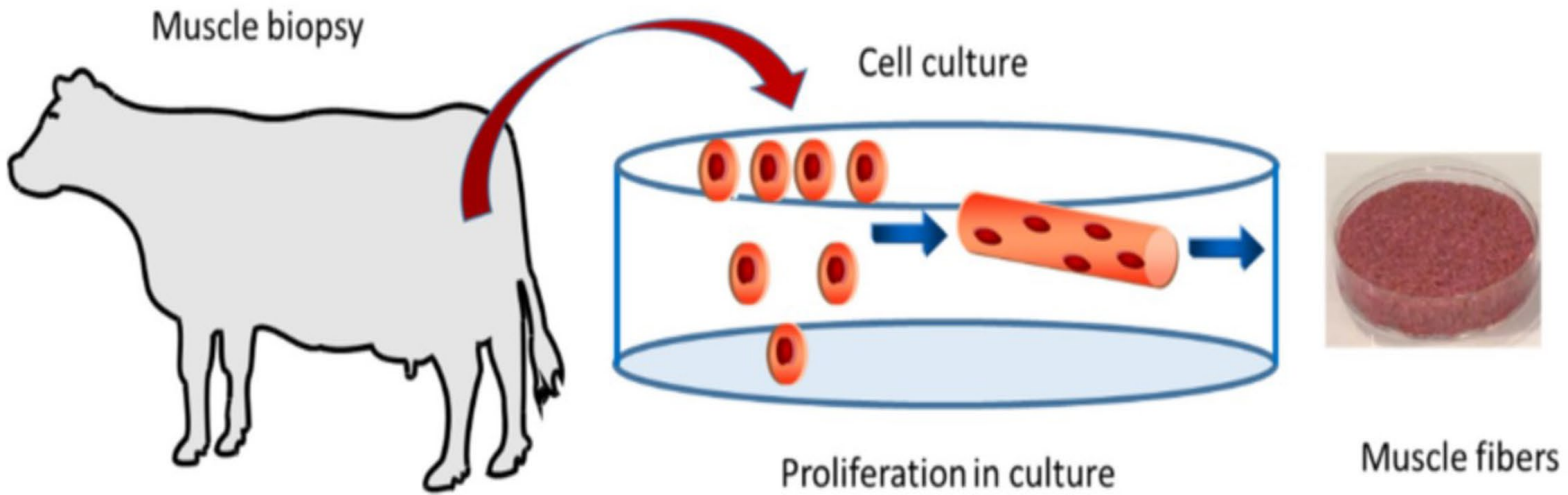
Introduction of “artificial meat” provided to respondents adapted from (23).

As an introduction to all the surveys, basic information on “artificial meat” was provided with a small text and an illustration to avoid confusion with any other type of “artificial meat,” e.g., from plant proteins (Figure 1). In at least one question, different wordings (such as “cell-based meat,” “cultured meat,” “lab meat,” etc) were used for better understanding and to avoid any bias in the answers. In this specific question, respondents were asked for the best wording of this new product.

The three surveys were organized around six sets of questions:

1. **Socio-demographic information:** this involved the collection of information such as gender, age, education level, area of work, net monthly income, meat consumption, and familiarity with “artificial meat” (Supplementary material - Questions 1 to 8).
2. **General questions:** These questions were asked as a preamble regarding the respondents’ food purchasing criteria and whether they had ever heard of this product (Supplementary material - Questions 9–10).
3. **Attitude towards societal challenges:** the objective here was to collect information on the respondents’ attitude towards the societal challenges facing conventionally produced meat (meat from conventionally raised farm animals) and “cell-based meat,” with regard to ethical, environmental, traditional meat industry, and rural life issues (Supplementary material - Questions 11 to 16).
4. **Characteristics of the product.** Questions were related to the perception of healthiness and eating quality of “artificial meat” (Supplementary material—Questions 17–18).
5. **Potential interests:** these questions aimed to evaluate consumer acceptance of “artificial meat.” They also had the aim to capture personal perceptions of this novel food compared to conventionally produced meat, including a question on how to name this product (Supplementary material—Questions 19 to 27).
6. **Development strategies:** the respondents were asked about future development strategies for the marketing of “cell-based meat” (Supplementary material—Questions 28 to 32).

Whereas the third survey included the six groups of questions, the survey 1 included the 4 first groups, and survey 2 the 2 last.

### Data collection

Respondents were randomly recruited using the KASI Insight platform. KASI Insight is an African-based consulting firm that conducts surveys for market research. Kasi insight implemented a computer assisted self-interviewing (CASI) process as its main methodology which is conducted at respective connected locations (businesses, community centers, etc.) which offer respondents access to complete the survey on a desktop, without incurring data costs. CASI is a technique for survey data collection in which the respondent uses a computer to complete on-line the survey questionnaire without an interviewer administering it to the respondent. This assumes the respondent can read well (enough) and understand either English or French.

A primary rationale for CASI is that some questions are so sensitive that if researchers hope to obtain an accurate answer, respondents must use a highly confidential method of responding.

The CASI process includes multiple quality controls, including trained interviewers available at the interview sites to assist respondents when they have questions. The CASI process also include: (1) Cross-checking and authentication: All completed surveys are checked and 20% of questionnaires are cross-checked. (2) Verification of data entry: validation and verification include predefined survey rules and matching processes.

Data were collected through monthly surveys. The target population for these surveys was the adult population of major urban cities in each of the participating countries. In most of these countries, this population is representative of the economically active population, and the main decision makers in household purchases. Rural residents were therefore excluded from the surveys.

Survey responses were voluntary. Each interview took an average of 15 to 20 min. Interviews were conducted in English and French. No quotas were imposed on the survey, allowing city residents of all demographics a fair chance to be included in the survey.

The first and second surveys were conducted in the main urban centers of 12 African countries: Cameroon (Yaoundé and Douala), Congo (Brazzaville), DRC Congo (Kinshasa), Ghana (Accra, Labadi, Teshie, Nunua, Kumasi), Ivory Coast (Abidjan, Yopougon, Angre, Abodo, Bouake, Williamsville), Kenya (Nairobi, Mombasa, Nakuru), Morocco (Rabat, Casablanca), Nigeria (Lagos, Abuja, Port harcourt, Abia), Senegal (Dakar), South Africa (Johannesburg, Cape Town), Tanzania (Dar Salaam, Arusha) and Tunisia (Tunis).

**Figure 2:**
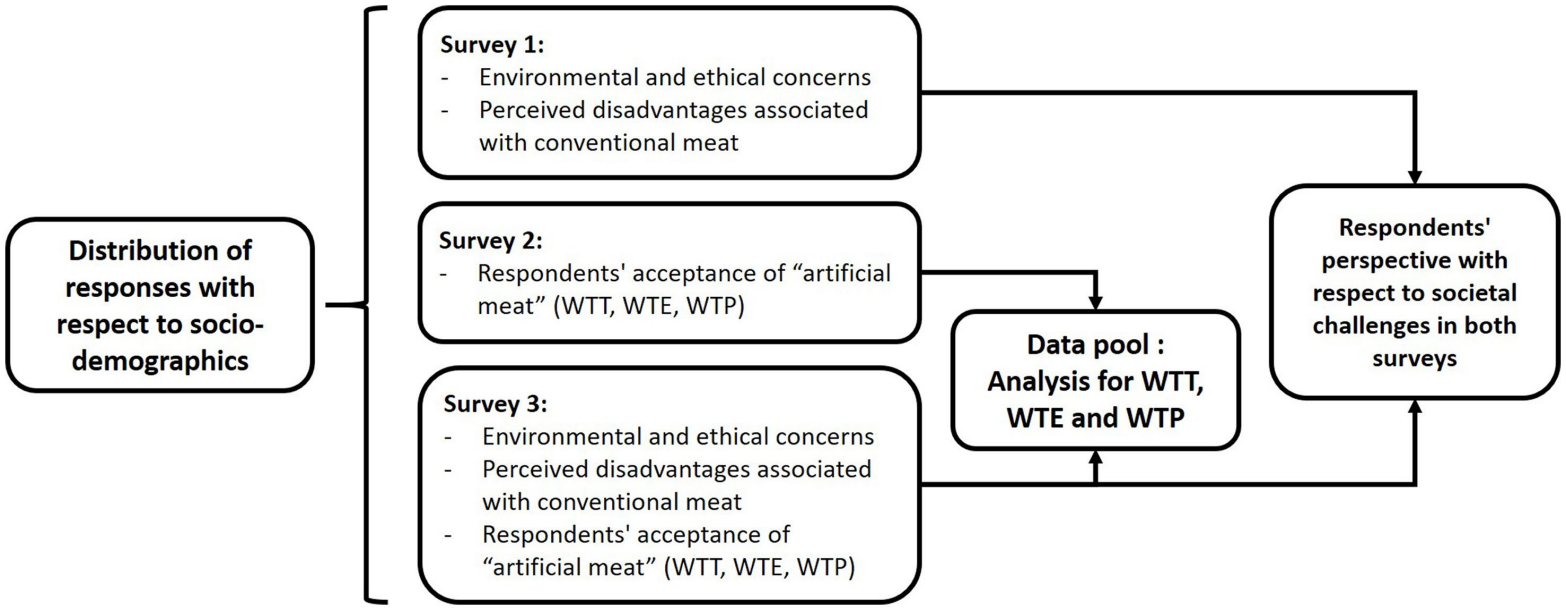
Survey process flow.

The complete survey process flow is shown in Figure 2. The first survey was constituted of 21 questions. This survey (Supplementary material-Questions 1 to 19, then 29 and 31) analyzed environmental and ethical concerns of the respondents (global warming, animal welfare, animal suffering, and slaughter) as well as the disadvantages associated with conventional meat as perceived by these respondents (limited agricultural resources and population growth).

The second survey (Supplementary material-Questions 1 to 7 and 20 to 32, excluding 29) analyzed respondents’ acceptance of “artificial meat” through their willingness to try (WTT), willingness to pay (WTP), and willingness to eat regularly (WTE).

The third survey was a combination on the first 2 surveys which was constituted of 33 questions. This survey analyzed both aspects (societal challenges and respondent acceptance) with fewer respondents (n = 1,111) from only 5 countries (Cameroon, Ghana, Kenya, Morocco, and South Africa) to represent different parts of Africa (Central, West, East, North, and South, respectively) and with the greatest potential to obtain more responses in a limited time period due to time constraints. The results of the two surveys were compared to assess the replicability of the methodology.

An average sample of 500 people per country was surveyed, targeting men and women over the age of 18. Because the population size of these sampled cities is generally greater than 500,000, the minimum recommended sample size for this population is 377, and we collected between 728 and 1,345 answers per country (for Cameroon, Ghana, Kenya, Morocco and South Africa) after pooling data from the first or second survey on one hand and from the third survey on the other hand in order to explore respondents’ opinions.

Pooling two or more cross-sectional survey data sets (i.e., stacking comparable data sets on top of one another) can serve different purposes including to increase the sample size in hopes of improving the precision of a point estimate. The latter purpose is especially common when making inferences on a subgroup, or domain, of the target population insufficiently represented by a single survey data set (26). Consequently, answers to questions numbers 25 to 28 related to WTT, WTE and WTP for 5 countries (Cameroun, Ghana, Kenya, Morocco and South Africa) were pooled to be analyzed together. For all other questions, results were compared between surveys without pooling to take into account data from the 12 countries from surveys 1 ad 2.

### Statistical analyses

The data were analyzed using R software (Version 4.0.2) (27). For most of the questions, respondents were asked to provide responses on a Likert-scale: strongly disagree (score 1), somewhat disagree (score 2), undecided (score 3), somewhat agree (score 4), and strongly agree (score 5), with the exception of a few qualitative variables which were then coded as quantitative variables to assess the influence of socio-demographic variables on WTT, WTP and WTE. The chi-2 test was used to compare the distribution of results between the two surveys.

WTT was coded quantitatively from “Definitely not” = 1 to “Definitely yes” = 5 and WTP: from “Much less” = 1 to “Much more” = 5. For WTE, the question was initially asked with multiple responses and was coded as “1” (for respondents who answered: In restaurants, At home, In prepackaged ready-to-eat meals, I do not want to eat “artificial meat” regularly, Other) and “2” (do not want to eat “artificial meat” regularly).

The initial investigations targeted the relationships between socio-demographic information and variables of willingness by means of ANOVA supplemented with a pairwise comparison between significant groups using Tukey HSD (by the R package “agricolae”). The one-way analysis of variance (ANOVA) was used to highlight any statistically significant differences between the means of the different groups corresponding to the levels of each factor of variation studied. The model was performed with the data collected from the second and third survey and for 5 different countries (Cameroon, Ghana, Kenya, Morocco and South Africa) with the different socio-demographic factors: y = Country + Gender + Age + Education + Income + Country × Gender + Country × Age + Country × Education + Country × Income + Gender × Age + Gender × Education + Gender + Income + Age × Education + Age × Income + Income × Age. ANOVA was run a second time with only the significant factors and significant interactions included in the model. Differences were considered significant at a value of *p* <0.05. Lsmeans (Least-Squares Means which are means that are computed based on a linear model such as ANOVA) were calculated in order to detect statistical differences across the different groups (28).

After performing the ANOVA, the effect size was investigated. Effect sizes could be used beyond significance tests (*p*-values), because they estimate the magnitude of effects, independent from sample size (29). The effect size was evaluated in R software (Version 4.0.2) with the package “sjstats.” The effect size was calculated by eta squared -denoted as η^2^. The eta squared corresponds to the total variability in the dependent variable accounted for by the variation in the independent variable. It is calculated as the ratio of the sum of squares for each group level to the total sum of squares. It can be interpreted as percentage of variance accounted for by a variable (30). The effect sizes are reported in the Supplementary material. Effect sizes varied between 0.01 and 0.02. for the different variables, which is considered to be low to moderate (30) (< 0.01 corresponds to a Small effect size, between 0.01 and 0.06 to a medium effect size and > 0.14 correspond to a large effect size).

In addition, a Principal Component Analysis (PCA) (by the R packages “FactoMineR” and “Factoextra”) was performed with the quantitative data of the third survey for which all responses from the same respondents were available to represent and model multidimensional point cloud surveys, showing whether relationships exist between the variables as previously done with similar data (19). PCA allows for the calculation of new variables, called principal components, which capture the variability in the data. This enables information to be described with fewer variables than originally present. The principal components are linear combinations of the original variables. The first principal component is the combination of variables that explains the greatest amount of variability in the data. The second and subsequent principal components describe the maximum amount of remaining variability and must be independent (orthogonal) between them and to the first principal component.

## Results

The sociodemographic characteristics of the respondents of the three surveys are detailed in Table 1. The result description follows the outline below:

– Comparison of socio demographics data between the 3 surveys (questions 1 to 8).
– Comparison of results between survey 1 (in 12 countries) and survey 3 (in 5 countries) concerning respondents’ perspective in relation to societal challenges (questions 11 to 16 in common between surveys 1 and 3).
– Comparison of results between survey 1 (in 12 countries) and survey 3 (in 5 countries) concerning the respondents’ potential interest in “artificial meat” (questions 19 to 27).
– Comparison of results between survey 2 (in 12 countries) and survey 3 (in 5 countries) (questions 19 to 31 in common between both surveys) concerning the potential acceptance of “artificial meat” by the respondents. Then, analysis of pooled results of WTT, WTE and WTP from 5 counties in common between surveys 2 and 3 (questions 25 to 28).
– Potential drivers of acceptance of “artificial meat” from data of survey 3 (answers to all questions from respondents of 5 countries).

**Table 1:** Distribution of responses with respect to socio-demographics.

Question	Response option	Survey 1		Survey 2		Survey 3	
		Number of responses	Percentage (%)^a^	Number of responses	Percentage (%)^a^	Number of responses	Percentage (%)^a^
Gender	Female	2,725	49.7	2,602	47.1	464	64,741.8
	Male	2,760	50.3	2,926	52.9	647	58.2
Age	18–30 years	1,583	28.9	1763	31.9	517	46.5
	31–50 years	3,745	68.3	3,593	65.0	544	49.0
	>51 years	157	2.8	172	3.1	50	4.5
Education	Primary school	216	4.0	205	3.7	27	2.4
	High school	412	7.5	410	7.4	52	4.7
	Undergraduate	149	2.7	168	3.1	25	2.25
	Technical Training	1,490	27.1	1,615	29.2	324	29.2
	Graduate	3,218	58.7	3,130	56.6	683	61.5
Monthly income	Under USD1,500	3,208	58.5	3,179	57.5	484	43.6
	More than USD1,500	2,277	41.5	2,349	42.5	627	56.4
Total		5,485	100	5,528	100	1,111	100

a Percentage of people who answered the questionnaire.

**Figure 3:**
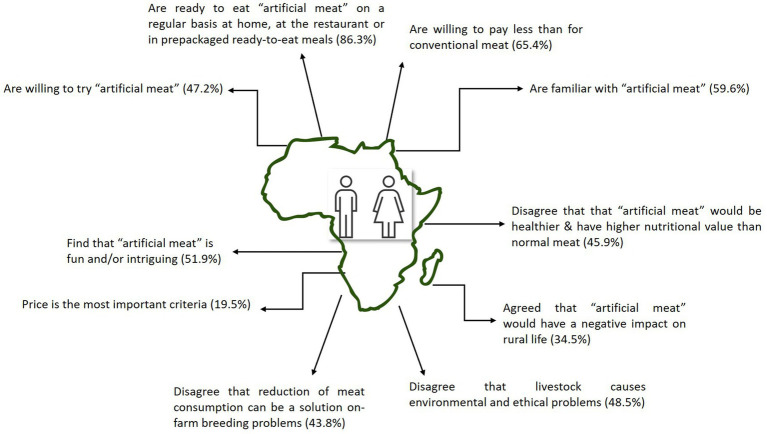
Respondents’ profile summary towards “artificial meat”.

A summary of the respondents’ profile and answers is shown in Figure 3.

### Socio-demographic data for all respondents

The first survey had 5,485 respondents, the second survey had 5,528 (the percentages of respondents per country in both surveys varied between 7 and 10%) and the 3^rd^ survey had 1,111 responses (the percentages of respondents per country varied between 19 and 20%). The socio-demographic characteristics of the respondents for all surveys are shown in Table 1.

All surveys had the same structure for gender and age with a majority being males representing 50.3, 52.9 and 58.2% of respondents in surveys 1, 2 and 3, respectively, and respondents between 31 and 50 years of age being the largest group (68.3%, 65%, and 49% respectively).

Most of the respondents were graduates in all surveys (58.7%, 56.6%, and 61.5% respectively) and had a monthly income of less than 1,500 USD (58.5%, 57.5%, and 43.6% respectively) (Table 1).

The respondents ate meat regularly, several times a week in in surveys 1 and 3 (38% in survey 1 and 38.2% in survey 3) and had heard of “artificial meat” (63.9% in survey 1 and 55.3% in survey 3) (data not shown).

In both surveys, most of the respondents were not scientists, and the majority did not work in the meat sector (54.9% in survey 1 and 50.5% in survey 3) (data not shown).

### Respondents’ perspective in relation to societal challenges

For all questions related to societal challenges (Table 2), no statistical difference was observed between the results of the two surveys which addressed these issues.

**Table 2:** Respondents’ perspective with respect to societal challenges in both survey 1 and survey 2.

Question		Response (1: completely disagree—5: completely agree)				
		1	2	3	4	5
In your opinion does on-farm breeding cause important environmental issues, e.g., huge water consumption and greenhouse gas emissions?	Survey 1	794 (14.5%)	1,579 (28.8%)	1727 (31.5%)	1,138 (20.7%)	246 (4.5%)
	Survey 2	221 (19.9%)	334 (30.1%)	370 (33.3%)	159 (14.3%)	27 (2.4%)
Do you believe that on-farm breeding can cause important ethical problems (e.g., animal suffering, animal slaughter)?	Survey 1	859 (15.7%)	1813 (33.1%)	1,527 (27.8%)	1,056 (19.3%)	229 (4.2%)
	Survey 2	301 (27.1%)	267 (25%)	376 (33.8%)	147 (13.2%)	20 (1.8%)
In your opinion, can the potential problems of on-farm breeding be dealt with by reducing our meat consumption?	Survey 1	735 (13.4%)	1,420 (25.9%)	1717 (31.3%)	1,314 (24%)	298 (5.4%)
	Survey 2	243 (21.9%)	293 (26.4%)	374 (33.7%)	179 (16.1%)	22 (2%)
Do you believe that if people ate Artificial meat instead of conventional meat, it would improve the welfare of animals and reduce animal suffering?	Survey 1	656 (12%)	1,246 (22.7%)	1790 (32.6%)	1,437 (26.2%)	355 (6.5%)
	Survey 2	252 (22.7%)	276 (24.8%)	383 (34.5%)	180 (16.2%)	20 (1.8%)
Using the following rating scale, do you think that Artificial meat could negatively impact livestock farming and the meat industry (e.g., by reducing the number of jobs available?)	Survey 1	547 (10%)	1,055 (19.2%)	1,599 (29.1%)	1,764 (32.2%)	519 (9.5%)
	Survey 2	239 (21.5%)	270 (24.3%)	343 (30.9%)	205 (18.4%)	54 (4.9%)
Do you think that Artificial meat would have a negative impact on rural life?	Survey 1	503 (9.2%)	1,098 (20%)	1,570 (28.6%)	1713 (31.2%)	600 (10.9%)
	Survey 2	201 (18.1%)	278 (25%)	332 (29.9%)	231 (20.8%)	69 (6.2%)
To what extent do you believe that Artificial meat would be healthier & have higher nutritional value than normal meat?	Survey 1	724 (13.2%)	1,291 (23.5%)	1913 (34.9%)	1,185 (21.6%)	371 (6.8%)
	Survey 2	292 (26.3%)	264 (23.8%)	397 (35.7%)	132 (11.8%)	26 (2.3%)
In your opinion do you believe that Artificial meat is tastier compared to normal meat.	Survey 1	1,032 (18.8%)	1,171 (21.3%)	1,898 (34.6%)	1,095 (20%)	288 (5.2%)
	Survey 2	284 (25.6%)	297 (26.7%)	399 (35.9%)	118 (10.6%)	13 (1.2%)
In your opinion would you say that you have emotional resistance to trying out Artificial meat (e.g., disgust or nervousness)?	Survey 1	548 (9.9%)	1,516 (27.4%)	2,197 (39.7%)	853 (15.4%)	414 (7.5%)
	Survey 2	294 (26.5%)	288 (25.9%)	371 (33.4%)	112 (10.1%)	46 (4.1%)

In surveys 1 and 3, most of the respondents disagreed with the idea that livestock can cause environmental problems (43.3% of responses were 1 and 2 on a scale of 0–5 point in survey 1 and 50% in survey 3) and ethical problems (48.8% in survey 1 and 52.1% in survey 3).

The respondents in both surveys also disagreed with the reduction of meat consumption as a solution to the problems caused by on-farm breeding (39.3% of responses were 1 or 2 in survey 1 and 48.3% in survey 3). The respondents disagreed on the fact that “artificial meat” would be healthier (36.7% of responses were 1 or 2 on scale of 0–5 in survey 1 and 55.1% in survey 3) and tastier (40.1% of responses were 1 or 2 on a scale of 0–5 in survey 1 or 52.3% in survey 3) than conventional meat. The respondents in both surveys agreed that “artificial meat” would have a negative impact on rural life (42.1% of responses were 4 or 5 in survey 1 and 27% in survey 3).

Some respondents expressed some emotional resistance to “artificial meat” in both surveys (22.9% of responses were 4 or 5 in survey 1 and 14.2% in survey 3) (Table 2).

### Potential interest in “artificial meat”

In both surveys, respondents considered price (18.8% in survey 1 and 20.1% in survey 3) as the most important criterion when purchasing meat, followed by meat quality (17.2% in survey 1 and 15.8% in survey 3) (Table 3). Some respondents in both surveys considered “artificial meat” as safe (14.2% in survey 1 and 18.1% in survey 3).

**Table 3:** Potential interest in “artificial meat” in both survey 2 and survey 3.

Question	Response options	Survey 1 and 2 (*n* = 11,013)		Survey 3 (*n* = 1,111)	
		Number of responses	Percentages (%)	Number of responses	Percentages (%)
Which of the following would you say are important considerations for you when you go to shop for meat? Which of the following would you say are important considerations for you when you go to shop for meat? (multiple choice question)	Ethics of how the meat was produced, e.g., were the animals allowed to roam freely	923	5.5	491	12.5
	Environmental impact of the food/meat during its production	1,572	9.4	501	12.7
	Price	3,150	18.8	792	20.1
	Quality of the meat (taste, juiciness, tenderness) …	2,884	17.2	624	15.9
	Appearance of the meat (e.g., its color, freshness)	2,220	13.3	483	12.3
Would you accept Artificial Meat as a viable alternative to normal meat in the future (Just like other meat substitutes like Soy proteins)?	Yes, I already eat meat substitutes or meat alternatives	837	15.3	278	25.1
	Yes, but I do not eat meat substitutes or meat alternatives	1,579	28.8	371	33.4
	No, but I eat meat and/or meat alternatives	1,804	32.9	286	25.7
	No, I do not eat meat substitutes and/or meat alternatives	1,265	23.1	176	15.8
Which of the following reasons would be most likely to persuade you to try Artificial meat?^a^	As a solution to feed the ever-growing human population	1,647	12.2	445	14.4
	It has more attractive pricing than conventional meat	1,646	12.2	509	16.4
	Ethics – it improves the wellbeing of animals and reduces animal slaughter	1,861	13.8	535	17.3
	Less risk of Zoonosis (disease that can be transmitted from animals to people, e.g., Foot & mouth disease)	1,630	12.1	462	14.9
	Attractiveness of high-tech technologies	1,371	10.1	330	10.6
	Curiosity	1,584	11.7	260	8.4
And which of the following would be the most likely reasons why you would not be willing to try Artificial meat?^a^	It is unnatural	1,756	12.7	47	20.0
	It is less tasty/appealing	1,587	11.4	15	6.4
	I am worried about its safety	1,929	13.9	29	12.3
	It is more expensive than normal meat	1,667	12.0	15	6.4
	I am reluctant (feel disgusted/nervous)	1,584	11.4	22	9.4
	It has a negative impact on local farmers	1,546	11.2	22	9.4
	Negative impact on local farmers & their jobs	1,426	10.3	28	11.9
	I do not trust laboratories and artificial meat start-up companies	966	7.0	37	15.7
Which of the following statements would you associate with Artificial meat?^a^	Adequate nutrition	1,241	9.0	468	13.5
	Tasty /tastes similar to real/normal meat	1,527	9.2	497	14.3
	Safety	1,923	14.0	626	18.1
	Less as a solution to feed the ever-growing human population	1,548	11.3	452	13.0
	It is less expensive or has better pricing than conventional meat	1,354	9.9	406	11.7
	It has a smaller environmental footprint	1,267	9.2	327	9.4
	Leads to the reduction of farming	1,161	8.5	264	7.6
	Leads to no farming	1,038	7.6	218	6.3
In which of the following cases would you be most likely to eat Artificial meat regularly?	At the restaurant	1,323	23.9	462	29.3
	At home	1,431	25.9	474	30.0
	In prepackaged ready-to-eat meals (e.g., lasagna…)	1,101	19.9	372	23.6
	I do not want to eat artificial meat regularly	821	14.8	141	8.9
	Other	852	15.4	129	8.2
Now that you have learnt a little bit more about Artificial meat, what do you think about it?	It is promising and / or acceptable	1,257	22.7	437	39.3
	It is fun and/or intriguing	3,262	59	497	44.7
	It is absurd and/or disgusting	1,009	18.2	177	15.9
Artificial meat is already available in some countries, when do you think artificial meat will be widely accepted?	In the short term – 1 to 5 years	1,208	21.8	408	36,7
	In the medium term—6 to 15 years	2,381	43.0	345	31.0
	In the long term—more than 16 years	1,124	20.3	261	23.5
	Never	815	14.7	97	8.7

a Only major answers are indicated.

When asked how they perceive “artificial meat,” respondents from both surveys followed the same pattern. Of the three options proposed, most consumers found it “fun and/or intriguing” in both surveys with 59.1% in the first survey and 44.7% in the third one. In the first survey, 22.7% found it “promising and/or acceptable” while 18.3% found it “absurd and/or disgusting.” In the third survey, the figures were 39.3% and 15.9%, respectively.

The largest proportion of respondents in survey 1 are consumers of both meat and meat alternatives and did not consider artificial meat as a viable meat alternative (they represent 32.9% in Survey 1 and 25.8% in Survey 3), while the largest proportion in survey 2 did not eat meat substitutes and considered “artificial meat” as a viable alternative (they represent 33.4% in survey 3 and 28.8% in survey 1, Table 3).

In both surveys, ethical concerns were the most likely to convince respondents to eat “artificial meat” (13.8% in survey 1 and 17.3% in survey 3), while safety concerns were a major reason why respondents were not willing to eat “artificial meat” (13.9% in survey 1 and 12.3% in Survey 3). In addition, a significant proportion of respondents found “artificial meat” to be unnatural (20% in survey 3 and 12.7% in survey 1, Table 3).

In both surveys, most respondents preferred to consume “artificial meat” at home (25.9% in survey 1 and 30.0% in survey 2), followed by restaurants (23.9% in survey 1 and 29.3% in survey 2) and pre-packaged ready-to-eat meat (19.9% in survey 1 and 23.6% in survey 3, Table 3).

Majority of the respondents preferred the name “artificial meat” (18%) (followed by “Clean Meat”: 13.66%; “Lab meat”: 13.36%; “Cultured Meat”: 13.22%; “Cellular Meat”: 11.13%; “*In-vitro* Meat”: 11.59%) and 61% agreed the product could be labelled as meat when commercialized, whereas 39% thought the opposite (data not shown).

### Potential acceptance of “artificial meat”

#### Willingness to try (WTT)

In surveys 2 and 3, many respondents were willing to try “artificial meat.” Indeed, in survey 2, 39.2% were willing to try “artificial meat” (8.9% definitely try and 30.3% probably try), 36.6% were unsure or not yet decided whether they would try “artificial meat” and 24.2% were not willing to try “artificial meat” (14.7% will probably not try, 9.5% will definitely not try). In survey 3, a majority (55.2%) is willing to try “artificial meat” (26.1% will definitely not, 29.1% will probably try) as in the first survey compared to 18.36% who are unwilling to try (4.7% definitely not, 13.7% probably not), with 26.5% of respondents being undecided (Table 3).

In survey 3, WTT was also significantly affected by the perceived impacts of conventional meat on livestock, rural life, the environment, ethics (*p* < 0.0001) and the interaction between the country and the frequency of meat consumption (*p* < 0.0001).

**Figure 4:**
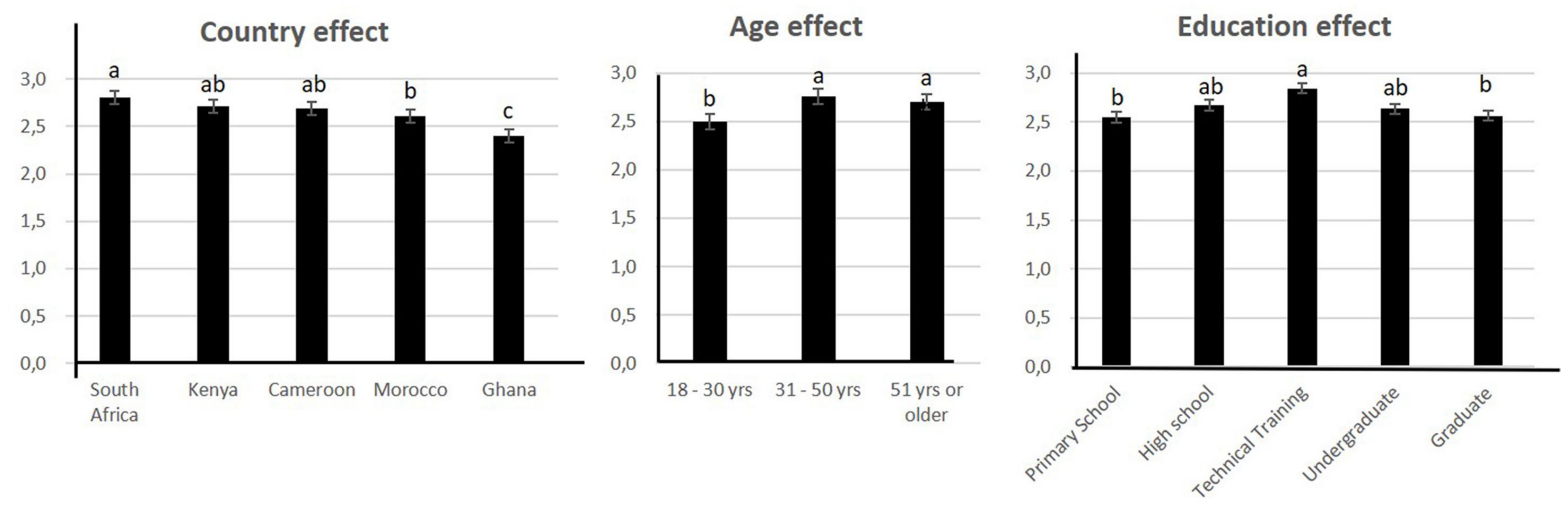
Significant factors affecting willingness to try “artificial meat.”

The pooled data for the 5 countries in common between surveys 2 and 3 was analyzed for 5 countries (Cameroun, Ghana, Kenya, Morocco and South Africa). The results showed that WTT differed significantly according to country, age, income and education (Figure 4; Table 1,). Interactions were significant between country and age, country and income, country and education, gender and income and education and income (Table 1).

South Africa came out as the country that was the most willing to try “artificial meat” while Ghana was the least willing to do so (Figure 4).

Those most willing to try were aged between 31 and 50 and had revenue less than $1,500 (data not shown). Respondents with a technical education were more willing to try (Figure 4).

#### Willingness to eat (WTE) regularly

Most respondents were willing to eat “artificial meat” on a regular basis (85.3%) in survey 2 as well as in survey 3 (87.22%).

In survey 3, WTE was also significantly affected by the perceived impacts of conventional meat on livestock (*p* < 0.0001), rural life (*p* < 0.0001), the environment (*p* < 0.006), ethics (*p* < 0.002) as well as the frequency of meat consumption (*p* < 0.004).

**Figure 5:**
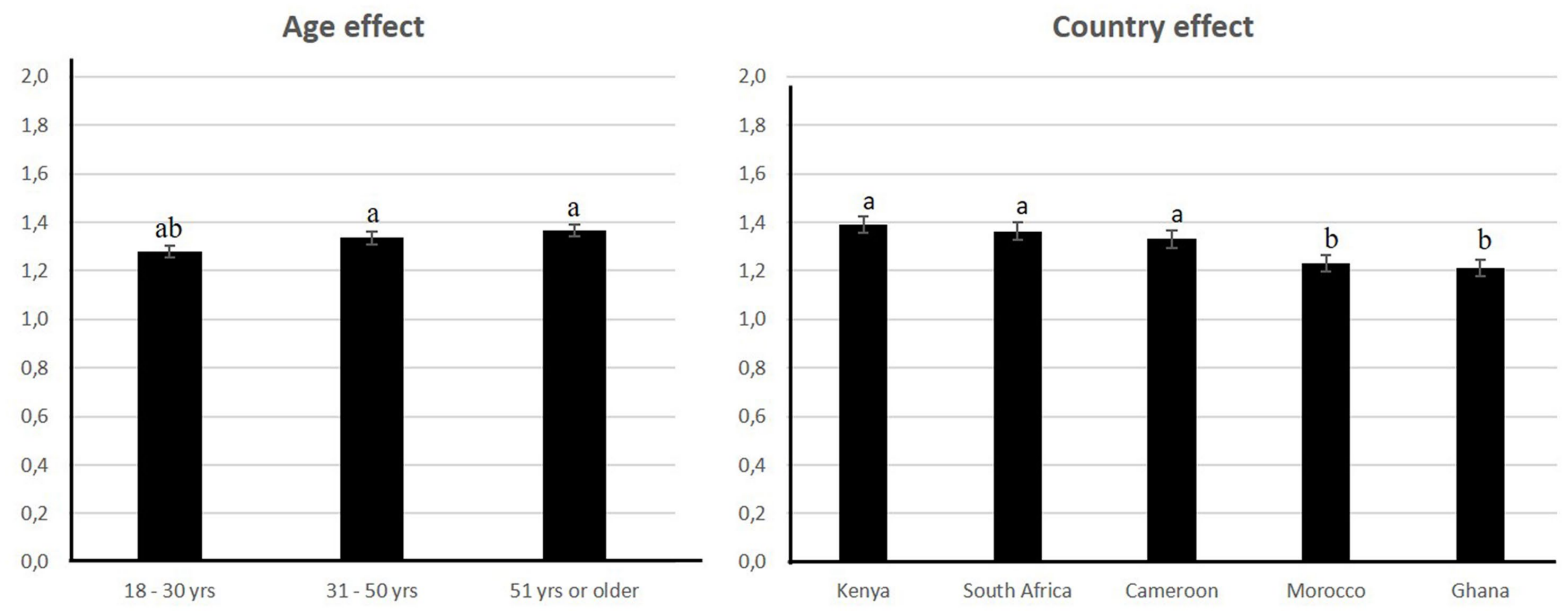
Significant factors affecting the willingness to eat “artificial meat.”

The pooled data for the 5 countries in common between survey 2 and 3 showed that WTE significantly according to country and age (Figure 5; Table 2). Interactions were significant between country and age, country and gender, country and income, country and education and education and income (Table 2).

Kenya was the most willing to eat “artificial meat” but did not differ significantly from South Africa and Cameroon (Figure 5).

Those most willing to eat were over 51 years of age but did differ significantly from respondents aged 31–50. WTE was higher for males with higher incomes and for females with low incomes ($3,000–4,000 and $4,000 or more and $1,500 or more for females). These 3 groups did not differ significantly. Respondents with a technical education were more willing to try.

#### Willingness to pay (WTP)

In survey 2, 60.7% of respondents were willing to pay less than for conventional meat (36.4% were willing to pay less and 24.3% were willing to pay much less than for conventional meat) while 12.7% were willing to pay more (8.6% more and 4.1% much more than for conventional meat) and 26.7% were willing to pay the same price as for conventional meat.

The same pattern was observed in survey 3, 70.0% of the respondents were willing to pay less than for conventional meat (36.4% were willing to pay less and 33.6% were willing to pay much less than for conventional meat) while 10.9% were willing to pay more (7.9% more and 3.0% much more than for conventional meat) while 19.1% were willing to pay the same price as for conventional meat.

**Figure 6:**
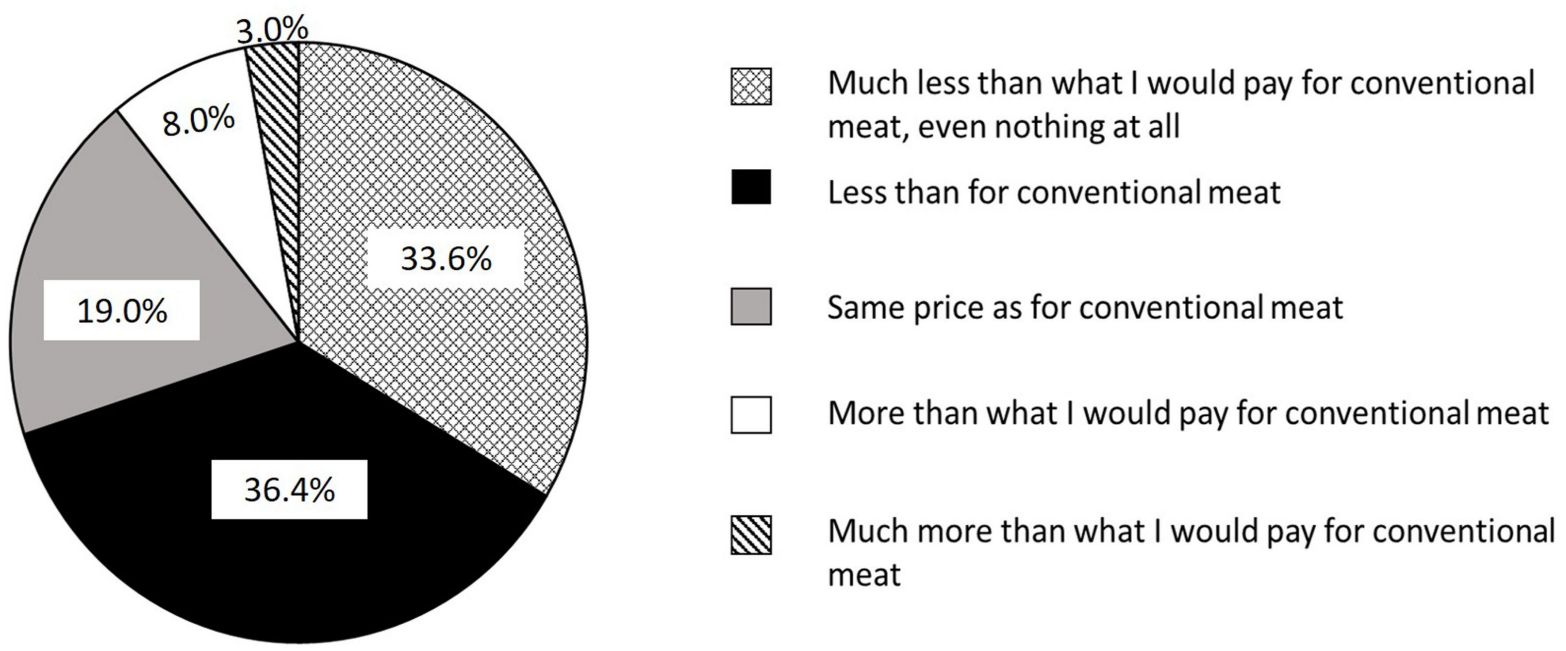
Percentages for willingness to pay for “artificial meat”.

Pooled results of the two surveys are indicated in Figure 6.

In survey 3, WTP was also significantly affected by the perceived impacts of conventional meat on livestock, rural life, the environment, ethics (*p* < 0.0001) as well as the frequency of meat consumption (*p* = 0.004).

**Figure 7:**
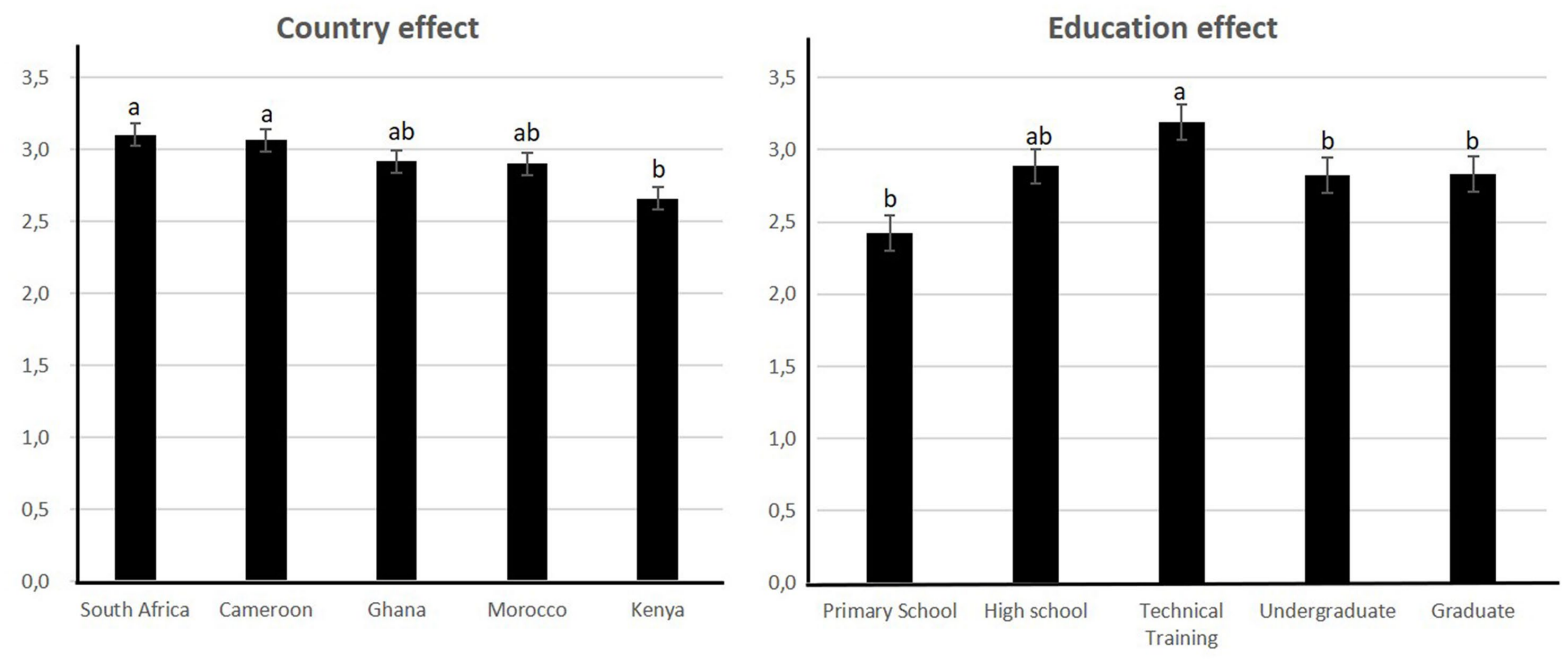
Significant factors affecting the willingness to pay for “artificial meat.”

Analyses of the data pool for 5 countries in common between survey 2 and 3 showed that WTP differed significantly according to country and education (Figure 7; Table 3). Interactions were significant between country and age, country and income, and education and income (Table 3).

The country that was the most willing to pay was South Africa and the least willing to pay was Kenya (Figure 7).

### Potential drivers of acceptance of “artificial meat”

**Figure 8:**
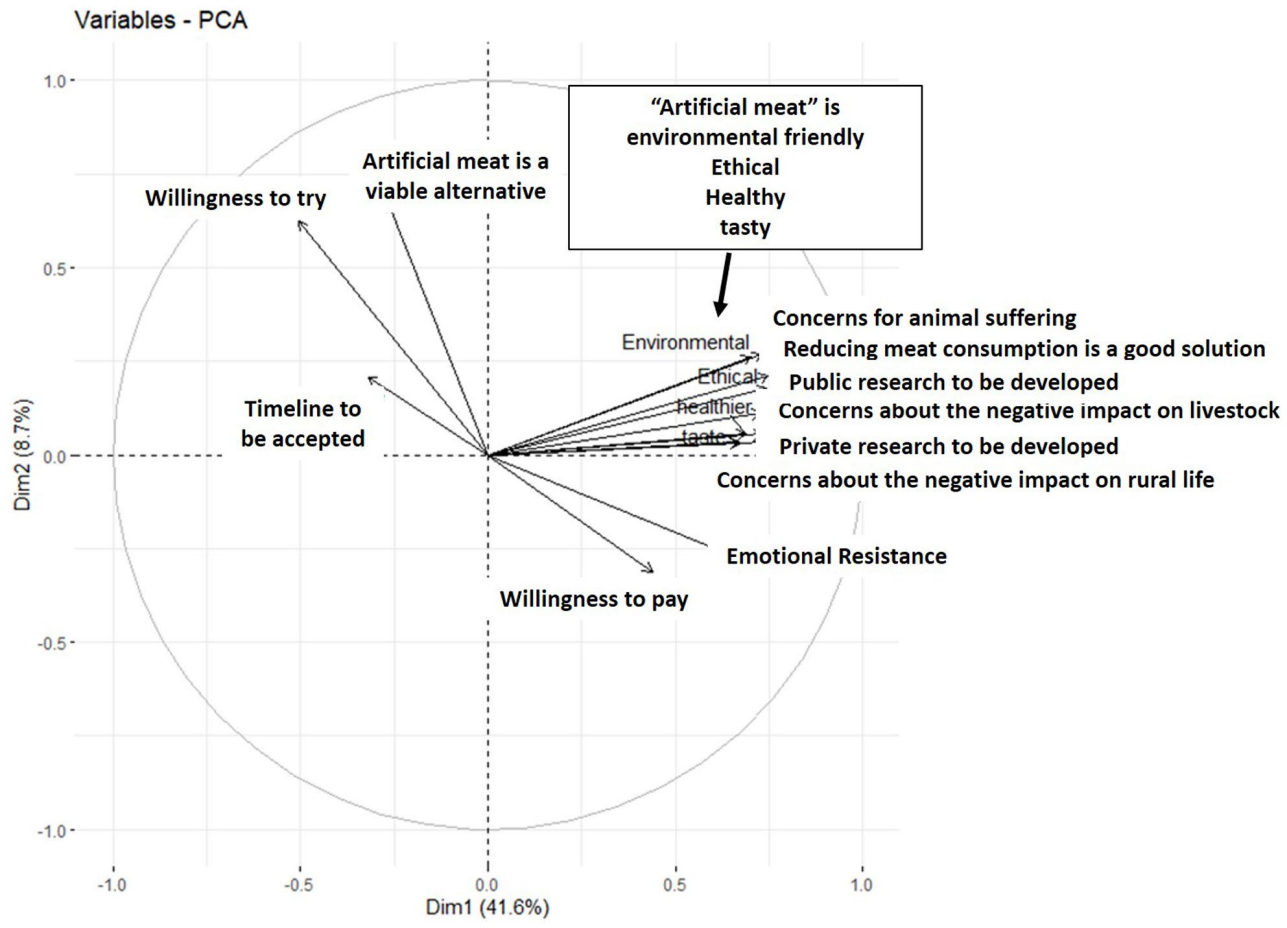
Principal Component Analysis of the main quantitative variables studied in survey 3.

A PCA was performed to investigate the underlying motives and barriers to acceptance of “artificial meat” by respondents, with all quantitative variables of survey 3 for which all responses from the same respondents were available (Figure 8) (12 questions).

The PCA revealed 2 groups of correlated variables on either side of the vertical axis. WTT was positively correlated with “artificial meat” perceived as a viable alternative to meat and its perceived timeline for availability. WTP was positively correlated with societal challenges (concerns about the ethics and environmental problems caused by on-farm breeding, animal suffering, impact on rural life and livestock) and negatively correlated with emotional resistance.

In this analysis, WTE was negatively correlated with WTT (r = −0.40; *p* < 0.001) but very slightly correlated to WTP (r = 0.04; *p* < 0.05). WTT was negatively correlated to WTP (r = −0.30; *p* < 0.001). Emotional resistance was negatively correlated with WTT (r = −0.50; *p* < 0.001) but positively correlated with WTE and WTP (r = 0.30 for both; *p* < 0.001).

## Discussion

### What factors will affect the acceptance of “artificial meat” across Africa?

The acceptance of “artificial meat” depends on several factors such as age, income and education.

Contrary to other studies which evidenced a gender effect (23–25, 31), in this study, gender did not have an effect on the acceptance (WTE & WTT) of “artificial meat,” as argued by other studies that did not find an association between gender and food neophobia (32–34). This contradiction could be explained by the fact that our survey was not fully representative of the African population in terms of urban and rural populations, with 58% of Sub-Saharan Africa being rural and only about 30% in North Africa (35, 36). In addition, in previous surveys, different interactions of the effect of gender with other effects have been observed, which explains why the effect of gender is not consistent (23, 37).

According to the PCA, WTP could be associated with positive acceptance of “artificial meat.” WTT was negatively correlated with WTP and WTE. This result is counterintuitive as one would expect WTT to be a precursor to WTE. This result may indicate that WTT may have different motives from WTP and WTE. The main motive for WTT would be curiosity (19), while WTP and WTE are motivated by ethics and environmental issues related to livestock, as well as price, as discussed below. This has also been observed in other studies that like in Taiwan where consumers are willing to buy plant-based meat alternatives for sustainable development of the environment despite their high prices. This study concluded that consumers’ perception of green value will affect their attitude toward green products (38, 39). It was also suggested that that consumers who are highly concerned about animal welfare and environmental issues are likely to consider livestock meat production as causing more ethical and environmental problems than their counterparts, which may affect negatively their consumption of meat or positively their consumption of meat alternatives (40).

Other factors that have affected African consumers’ perspective on “artificial meat” are also discussed.

#### Age

Age was the most important factor in our study. Other studies have shown that “artificial meat” is more attractive to younger respondents in France, Brazil and Germany (19, 23, 41). In our study, older respondents (>30 years) were the most willing to accept (WTT & WTE) “artificial meat” but age had no influence on WTP. This is in line with results in China where young respondents had the lowest WTT (25). This is also in line with the Australian survey of the Generation Z (18–24 years of age) who feared betraying Australia as a meat-eating nation (42). In Africa, livestock entrepreneurship is a self-employment option where unemployment rates are still high for younger populations (43). However, these results can be mitigated because our survey did not represent younger respondents very well. Indeed, 40% of the African population is under 15 years of age (44) and this age group was not included in the survey.

#### Country

Our survey suggests that although there is a general positive outlook on “artificial meat,” the extent of this potential acceptance is not the same in all countries. This indicates the diversity of African consumers in terms of food behavior, income, gender, age and education, as all of these differ in these countries. In our study, consumers from South Africa were the most willing to try and pay for “artificial meat,” with no difference to Kenyan consumers who were the most willing to eat. In a previous study that surveyed 10 countries, South Africa was shown to have a high level of acceptance of “artificial meat,” second only to Mexico (21). The suggested reason by this study was that South Africa has been influenced from other countries such as UK or the Netherlands. This observation may also be due to the various migrants in the country. Indeed, South Africa has the highest number of migrants in its population, followed by Kenya, Cameroon, Ghana and Morocco (45, 46). The fact that Ghana and Morocco have the least migrant populations could explain why they are the least accepting of “artificial meat.”

These countries with a higher percentage of migrant population are therefore more open to novel foods and will consequently have higher levels of acceptance of “artificial meat.” South Africa differs from a number of other African countries due to the strong influence of European cuisine (21). The migrant population from other countries may differ between South Africa and other African countries. Indeed, African immigration is mostly characterized by movements within the continent (47), with the exception of South Africa which has had a more western migrant population compared to other African countries (21).

Added to this, South Africa is home to the first company to produce “artificial meat” on the African continent and this company has been funded by overseas investors as well as South Africans themselves. Kenya appears to be the country the most willing to eat “artificial meat” due to its forefront position in relation to drought (48).

Otherwise, Kenya is currently losing its livestock due to drought and may perceive “artificial meat” as a source of relief for its meat consumption but is less willing to pay for “artificial meat.” Ghana appears to be less willing to accept “artificial meat.” “Artificial meat” companies must therefore have a country-specific strategy, targeting those with similar demographics and food consumption habits.

#### Income

Income as well as purchasing power in Africa are increasing, but despite this, respondents are still willing to pay less for “artificial meat” than for meat. In the pooled data, most respondents had a monthly income of more than USD 1,500 (43.56% < USD 1,500 and 53.44% > USD 1,500). The average salary in Africa is approximately USD 758 with great variability between countries (49–51) but the price of beef is much lower than in European countries, which means that purchasing power should be adjusted accordingly. The price of beef in the countries surveyed varies from USD 4 to USD 13 (52). The African Development Bank (AfDB) classification measures the middle class as people living on an income of between USD 2 and 20 per person per day, or USD 60 and USD 600 per month (2011) (53). This difference between countries was also reflected by the interaction effect between income and country in the ANOVA model.

Therefore, with this information, our survey only discriminated between rich and non-rich people. Despite this limitation, this can be considered as an indication that the WTP for “artificial meat “of African countries might be very low and, in any case, lower than for conventional meat.

This could lead to the conclusion that “artificial meat” will not be considered a premium product. A report from the consulting firm Mckinsey (54) indicated that “artificial meat” will initially bear a premium price tag, therefore putting it out of reach of some consumers, especially African consumers. Prices are likely to fall as the industry scales up, therefore pushing it on the spectrum of the long term (55). Most respondents consider “artificial meat” will be available in the medium term (6–15 years), as do the French (40.6%) and the Chinese (45.1%) respondents, but different from Brazil where 38.9% of respondents consider that “artificial meat” will be available in the long term (> 15 years).

#### Education

The literature has shown that education is a predictor of “artificial meat” acceptance since this product is more appealing to more educated consumers (56–58). Those with lower levels of education are more likely to have food neophobia (59). There has been an increase in the education level in Africa (60) but despite this increase, inequalities are still a critical issue (61). On average, only 9% of Africans were indeed enrolled in post-secondary education in 2019 (62) compared to 73% in Europe (62). In the cities of countries studied in this work, these proportions are 14% in Cameroon, 10% in Kenya, 17% in Ghana, 24% South Africa and 41% in Morocco. The respondents of our survey therefore represent only a minority of actual African citizens, as most of them have higher education.

Our survey showed that “artificial meat” is more appealing to respondents with a technical background. The reason for this might be that respondents with a technical background are more open to technological innovations. Our survey did not include non-educated respondents. Some experts believe that meat alternatives will create new job opportunities along the production chain (63–65). Others discussed the possibility of using plant ingredients as culture media instead of bovine serum (64). Some African countries could therefore become a supplier to this industry. On the other hand, these new technologies will require more trained and qualified staff. Education will therefore play a crucial role in ensuring that “artificial meat” is produced on the African continent. Animal farmers, crop-growing farmers and the rural community are indeed not generally highly educated and may suffer income losses and job losses as a result of the transition to more urban meat production, as they may not be qualified (63).

Education also had an interaction effect with income on WTP and WTE. This is because education is more accessible to those with higher incomes, especially in some developing countries.

### Acceptance of “artificial meat”

Most of the respondents in Brazil, France, China (19, 23, 25) and Africa (this study) were willing to pay less for “artificial meat” than for conventional meat but the percentage of African citizens was lower compared to the situation in the other countries (71%, 69%, and 87%, respectively, and 65% for Africa).

Overall, our results show that there may be a positive perspective on “artificial meat” in Africa. However, with the same questionnaire and methodology, WTT was lower than for Brazil, France and China (50%, 51%, and 66%, respectively, *vs* 47.2% on average in Africa) (19, 23, 25) but WTE was much higher (> 80%). This could be due to the fact that African countries found “artificial meat” more fun and/or intriguing than other countries (35%, 23%, and 49%, respectively, and 52% for Africa) (19, 23, 25). It appears that African consumers are keen to adopt “artificial meat” in their diets, and therefore it makes less sense to simply try it.

Interestingly, African and Chinese consumers show less emotional resistance (18.6 and 16.1%) compared to French and Brazilian respondents (55.5% and 32.4%) (23, 25). This may be explained by the fact that African and Chinese consumers are more open-minded and have more diverse dietary habits, such as eating insects (as in Africa) or plant proteins (as in China).

WTT was negatively correlated with WTP and was not associated with prospects for societal changes. This negative correlation suggests that African consumers who are ready to try “artificial meat” may not be willing to pay a high price for this product.

### How can “artificial meat” pave its way in a diverse continent?

Africa is vast and diverse. Consumers do not have the same expectations. “artificial meat” industries must therefore have strategies tailored to each country or at least to the five commonly known subregions: North or Northern Africa, West Africa, Central or Middle Africa, East Africa, and Southern Africa.

#### Price

Africans consider price as the most important factor when it comes to purchasing meat.

Price is considered to be a common negative factor for “artificial meat” (16). This could lead to consumer reluctance if “artificial meat” prices are high. This is why, in both surveys, respondents were willing to pay less than for conventional meat. In order to gain more mainstream recognition, “artificial meat” must be cheaper and possibly produce high quality cuts at a comparatively lower price, as indicated by (66). In practice, price competitiveness is therefore an important factor to take into consideration for African countries at least. Price does not only pose a problem on the consumption side but also on the production side since production costs are still high (67).

Another important point to be considered is how “artificial meat” will be made available to African consumers. Two scenarios can be hypothesized. The first is based on the idea that “artificial meat” would be imported from developed countries, with the exception of South Africa which has already started to produce “artificial meat.” The second scenario implies that African countries would have to produce “artificial meat” themselves. Both these scenarios raise certain issues. First of all, it might be more expensive to import “artificial meat” because of its current high price and limited production compared to conventional meat. Secondly, it could be more efficient or less expensive for African governments to implement better policies by helping local farmers rather than building new “artificial meat” industries.

African consumers may be willing to try “artificial meat” because they suppose it will be cheaper than conventional meat, since price is the primary driver in purchasing meat. Even though they are less willing to try “artificial meat,” they would be prepared to eat it regularly if the price was affordable. Furthermore, unlike in developed countries, Africans are unsure about the effects of livestock on the environment and ethical issues, which explains why price is by far the most important driver of “artificial meat” acceptance.

#### Safety

Most African respondents associated “artificial meat” with safety, like Chinese consumers (25). That is, they consider “artificial meat” as a safe product. This is because livestock has been associated with several diseases since in many countries there are no refrigerated facilities to store meat in safe conditions. In Africa, the number of zoonotic disease outbreaks increased between2012 and 2022 (68). The safety of animal-sourced food is therefore becoming a major concern for local populations and policy makers. However, there are still several uncertainties and limited scientific literature on the safety of “artificial meat” (15, 69).

The emphasis on safety may also stem from the 2014–2016 Ebola outbreak and the global COVID-19 pandemic. This pandemic helped raise awareness of the risk of emerging diseases related to animal exploitation. Africans could therefore perceive “artificial meat” as a way to limit these outbreaks in a context of weak health systems. However, there are still some doubts as to the safety of “artificial meat,” such as the potential addition of chemicals to the culture medium, resulting in a “chemical” meat with potential negative consequences on consumer acceptance (67, 70). Deregulation of cell lines may also occur (as in cancer cells), which may lead to unknown effects on human health (67).

### Contributions and limitations of this work and future research

The answers to the questions in any survey are likely to depend on the wording and sequencing of the questions, which may influence the conclusions of the survey. Many studies have also been conducted by “artificial meat” promoters with clear objectives to influence consumers or accelerate marketing. However, our results could still be relevant to providing useful information for understanding the drivers or barriers to “artificial meat” acceptance by African’s rapidly urbanizing citizens. More relevant information could be derived from comparing results obtained with the same experimental design across countries, such as France (19), Brazil (23) and China (25), or across similar social groups (within the same study as in this work). More importantly, relevant studies on “artificial meat” are rare in Africa which makes contextual interpretation difficult.

In addition, there is clearly a strong generational effect, particularly with regard to ethical and environmental issues of livestock production, meat consumption, and therefore the overall perception of meat substitutes, including “artificial meat.” Moreover, young, well-educated people are likely to have a clearer idea of the potential and limits of science. All these facts may explain why older people may reject “artificial meat” less as shown in this work, or more, as shown in Germany and France (41), and in Europe (71). Consequently, targeting young, well-educated people as in this survey may better indicate the trends of “artificial meat” acceptance in the future, while underestimating or overestimating this potential acceptance at present.

Generally speaking, opinion surveys must be interpreted appropriately because to their limited representative character. However, this drawback can be partially offset by the consequent size of the sample and the duplication of our survey, which makes it possible to identify the major factors likely to explain, at least in part, the variability in WTT, WTE and WTP for “artificial meat,” and to analyze different segments of the population.

Finally, due to the importance of implicit attitudes that are difficult to capture, a recent survey illustrated the inadequacy of relying on self-reported measures when seeking to capture consumer opinions on unfamiliar products such as “artificial meat” (72). However, again, despite these limitations, comparing results obtained with the same experimental design between countries or between similar social groups (within the same study as in this work) is likely to provide useful information.

## Conclusion

Meat consumption in Africa is set to increase due to population growth, urbanization and income. This high demand will put pressure on a livestock industry that is not yet efficient in terms of productivity. Despite the small sample sizes of this survey, insights could still be gleaned from the results to get a broad view of the cultured meat perspective in the countries studied. As African countries vary in terms of consumer behavior, culture, tradition and demographics, the potential perception of the challenges facing livestock production and meat consumption is likely to vary accordingly. A strategy must therefore be tailored to each country. Price will be one of the main drivers in acceptance of “artificial meat” by several African countries. Therefore, policy makers, governments and local investors will have to make a choice to direct their financial help towards supporting more efficient meat production in order to meet the increasing demand of meat. Several options could be considered; investing in making livestock production more efficient and sustainable, investing in other animal protein alternatives such as protein from insects which are already widely consumed in many African countries, or investing in the production of meat alternatives such as plant-based meat or “artificial meat.” This choice will be reinforced by Africa’s limited investment capacity compared to richer countries.

## Data availability statement

The raw data supporting the conclusions of this article will be made available by the authors, without undue reservation.

## Ethics statement

Ethical review and approval was not required for the study on human participants in accordance with the local legislation and institutional requirements. The patients/participants provided their written informed consent to participate in this study.

## Author contributions

MK: survey, data analysis and draft writing. SC, M-PE-O, JL and J-FH: survey design. J-FH: surpervision. All authors contributed to the article and approved the submitted version.

## Conflict of interest

The authors declare that the research was conducted in the absence of any commercial or financial relationships that could be construed as a potential conflict of interest.

## Publisher’s note

All claims expressed in this article are solely those of the authors and do not necessarily represent those of their affiliated organizations, or those of the publisher, the editors and the reviewers. Any product that may be evaluated in this article, or claim that may be made by its manufacturer, is not guaranteed or endorsed by the publisher.
