# Supplementary material for: Consumer perception of “artificial meat” in the educated young and urban population of Africa
Source: Front Nutr. 2023 Apr 14;10:1127655. doi: 10.3389/fnut.2023.1127655 (PMC10140314; doi:10.3389/fnut.2023.1127655)
Supplement: Supplementary file 1 [file Data_Sheet_1.PDF]

* Required Information

Thank you for agreeing to take part in our survey to understand more about the Artificial meat. Please be assured that, all the information you share will be treated as strictly confidential.
Please read each question carefully and provide the answer that you feel most accurately describes your situation.

Thank you for your participation!

Merci d'avoir accepté de participer à notre enquête visant à mieux comprendre la viande artificielle. Soyez assuré que toutes les informations que vous nous communiquerez seront traitées de manière strictement confidentielle.
Veuillez lire attentivement chaque question et fournir la réponse qui, selon vous, décrit le mieux votre situation.

Nous vous remercions de votre participation !

* **1. D1. Gender**

Sexe

(Select one option)

- ◯ Male / Homme
- ◯ Female / Femme

* **3. D3. Please select the age group you fall under**
Veuillez sélectionner le groupe d'âge auquel vous appartenez **(Select one option)**

- ◯ 18 - 30yrs / 18 - 30 ans
- ◯ 31 - 50yrs/ 31 - 50ans
- ◯ 51 yrs or older/ 51 ans ou plus

* **4.** **D4. What's your highest level of education?**

Quelle est votre niveau d’éducation?

(Select one option)

- ○ Primary School / Ecole primaire
- ○ High school, no diploma / BEPC
- ○ High school diploma / Baccalauréat
- ○ Trade/technical/vocational training / Diplome technique
- ○ Bachelor’s degree / Licence
- ○ Master's degree / Maitrise (DEA)
- ○ Doctorate degree / Doctorat
- ○ Currently studying at tertiary level e.g. University/ Étudie actuellement au niveau tertiaire, par ex. Université
- ○ Professional Qualification e.g. CIMA / Qualification professionelle (ex. CFA)

* **5.** **D5. What religion do you belong to or identify with the most?**
À quelle religion appartenez-vous ou vous identifiez-vous le plus ? **(Select one option)**

- ◯ Traditional African religion (e.g. includes ancestral worship)/ Religion africaine traditionnelle (par exemple, comprend le culte ancestral)
- ◯ Hindu/ hindou
- ◯ Jewish/ juif
- ◯ Muslim/ musulman
- ◯ Catholic Christian / Chrétien catholique
- ◯ Other Christians e.g. Methodist, Baptist, Pentecostal denominations) /D'autres chrétiens, par ex. dénominations méthodiste, baptiste, pentecôtiste)
- ◯ I am not religious / je ne suis pas religieux
- ◯ (Other religions) ___________

* **6.** **D6. Please make sure you select your country of your residence ONLY to continue**

Veuillez vous assurer de sélectionner UNIQUEMENT votre pays de résidence pour continuer

(Select one option)

- ○ Ghana
- ○ Kenya
- ○ Cameroon
- ○ Morocco
- ○ South Africa

NOTE : IF ANSWER TO **Q6** is
Ghana Go to Page No. 8
Kenya Go to Page No. 7
Cameroon Go to Page No. 10
Morocco Go to Page No. 11
South Africa Go to Page No. 9

* **7.** **6. Which of these describes your personal monthly income?** ***Please tick the one option that most applies to you***
(Select one option)

- ○ Under KES165,000
- ○ Between KES165,001 to KES200,000
- ○ Between KES200,001 to KES275,000
- ○ Between KES275,001 to KES330,000
- ○ Between KES330,001 to KES440,000
- ○ More than KES440,000

NOTE : IF ANSWER TO **Q7** is
Under KES165,000 Go to Page No. 12
Between KES165,001 to KES200,000 Go to Page No. 12
Between KES200,001 to KES275,000 Go to Page No. 12
Between KES275,001 to KES330,000 Go to Page No. 12
Between KES330,001 to KES440,000 Go to Page No. 12
More than KES440,000 Go to Page No. 8

* **8.** **6. Which of these describes your personal monthly income?.** ***Please tick the one option that most applies to you*** **(Select one option)**

- ○ Under GHS9,000
- ○ Between GHS9,001 to GHS12,000
- ○ Between GHS12,001 to GHS15,000
- ○ Between GHS15,001 to GHS18,000
- ○ Between GHS18,001 to GHS24,200
- ○ More than GHS24,200

NOTE : IF ANSWER TO **Q8** is
Under GHS9,000 Go to Page No. 12
Between GHS9,001 to GHS12,000 Go to Page No. 12
Between GHS12,001 to GHS15,000 Go to Page No. 12
Between GHS15,001 to GHS18,000 Go to Page No. 12
Between GHS18,001 to GHS24,200 Go to Page No. 12
More than GHS24,200 Go to Page No. 12

* **9.** **6. Which of these describes your personal monthly income?.** ***Please tick the one option that most applies to you*** **(Select one option)**

- ◯ Under R22,500
- ◯ Between R22,501 to R30,000
- ◯ Between R30,001 to R37,500
- ◯ Between R37,501 to R45,000
- ◯ Between R45,001 to R60,000
- ◯ More than R60,000

NOTE : IF ANSWER TO **Q9** is
Under R22,500 Go to Page No. 12
Between R22,501 to R30,000 Go to Page No. 12
Between R30,001 to R37,500 Go to Page No. 12
Between R37,501 to R45,000 Go to Page No. 12
Between R45,001 to R60,000 Go to Page No. 12
More than R60,000 Go to Page No. 12

* **10.** **6. Lequel de ces énoncés décrit votre revenu mensuel personnel?** ***Veuillez cocher la seule option qui s'applique le plus à vous*** **(Select one option)**

- ○ Moins que XAF835,000
- ○ Entre XAF835,001 à XAF1,115,000
- ○ Entre XAF1,115,001 à XAF1,400,000
- ○ Entre XAF1, 400,001 à XAF1,700,000
- ○ Entre XAF1, 700,001 à XAF2,2300,000
- ○ Plus de XAF2,230,000

NOTE : IF ANSWER TO **Q10** is
Moins que XAF835,000 Go to Page No. 39
Entre XAF835,001 à XAF1,115,000 Go to Page No. 39
Entre XAF1,115,001 à XAF1,400,000 Go to Page No. 39
Entre XAF1, 400,001 à XAF1,700,000 Go to Page No. 39
Entre XAF1, 700,001 à XAF2,2300,000 Go to Page No. 39
Plus de XAF2,230,000 Go to Page No. 39

* **11. 6. Lequel de ces énoncés décrit votre revenu mensuel personnel?.** ***Veuillez cocher la seule option qui s'applique le plus à vous*** **(Select one option)**

- ○ Moins que MAD 13,500
- ○ Entre MAD 13,501 à MAD 17,900
- ○ Entre MAD 17,901 à MAD 22,500
- ○ Entre MAD 22,501 à MAD 27,000
- ○ Entre MAD 27,001 à MAD 36,000
- ○ Plus de MAD 36,000

NOTE : IF ANSWER TO **Q11** is
Moins que MAD 13,500 Go to Page No. 39
Entre MAD 13,501 à MAD 17,900 Go to Page No. 39
Entre MAD 17,901 à MAD 22,500 Go to Page No. 39
Entre MAD 22,501 à MAD 27,000 Go to Page No. 39
Entre MAD 27,001 à MAD 36,000 Go to Page No. 39
Plus de MAD 36,000 Go to Page No. 39

Artificial meat, is also known as in vitro meat, cultured meat, lab meat, clean meat, or synthetic meat. It is lab-grown meat, where the "meat" is grown outside the animal's body, using stem cells from the animal muscles (refer to the picture below). So it allows us to get meat without having to slaughter cows or other animals. There is a lot of interest in artificial meat as it can help solve world hunger by providing food for the growing human population. It will also help solve the increasing environmental concerns (e.g. global warming) and other ethical problems because it improves the lives of animals due to less slaughtering. For these reasons Scientific research is devoted to developing artificial meat as a viable source of meat for the future.

This research study is being conducted by French researchers from INRAE (Theix, 63), ISARA (Lyon, 69) and Bordeaux Sciences-Agro (Bordeaux, 33). The purpose of this survey is to understand how ordinary people like yourself feel about this new biotechnology and to find out what your preferences and concerns are. This is an interesting subject, and it should not take more than 15 minutes for you to complete. If you have any questions, you can write to the research team at 'futurecellmeat@gmail.com'.

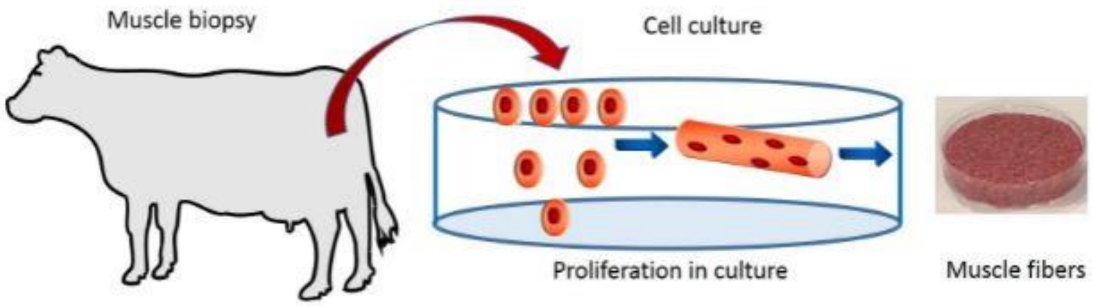

* **12.** **7. How are you involved in the Meat sector?** ***Please tick the one option that most applies to you*** **(Select one option)**

- ◯ I am a scientist, who works in the meat sector
- ◯ I am a scientist, who works outside the meat sector
- ◯ I am not a scientist, and I work in the meat sector
- ◯ I am not a scientist, and I do not work in the meat sector

* **13.** **8. How often do you eat meat?** ***Please tick the one option that most applies to you*** **(Select one option)**

- ◯ I never eat meat –I am vegetarian or vegan
- ◯ I rarely eat meat – once a week or less
- ◯ I regularly eat meat – several times a week
- ◯ I eat meat daily - or within each meal

* **14. 9. Before today, had you ever heard about Artificial Meat?** ***Please tick the one option that most applies to you*** **(Select one option)**

- ○ Yes
- ○ No

* **15.** **10. Which of the following would you say are important considerations for you when you go to shop for meat?** ***Please tick ALL the options that most applies to you***

- ☐ Ethics of how the meat was produced, e.g. were the animals allowed to roam freely
- ☐ Environmental impact of the food/meat during its production
- ☐ Price
- ☐ Quality of the meat (taste, juiciness, tenderness) …
- ☐ Appearance of the meat (e.g. its colour, freshness)
- ☐ Energy intake of the animal during production (Calories used)
- ☐ Nutritional value of the meat (e.g. No. of proteins, vitamins)
- ☐ Origins and how traceable it is to find out where the meat came from
- ☐ Safety
- ☐ Information on the nutrition labels, food labels or marks
- ☐ Others

SOCIETAL CHALLENGES

We will now look at your perceptions towards how meat is produced, with a focus on On-Farm Animal breeding. On Farm Animal Breeding is the selective mating of animals to increase the possibility of obtaining certain desired characteristics in the animal's offspring. For each answer, scale is from 1 to 5 where 1 means You completely disagree, and 5 means you completely agree.

* **16.** **11. Do you believe that on-farm breeding can cause important ETHICAL problems, (e.g. the animals suffering, the animals being slaughtered)?** ***Please tick the one option that most applies to you***

(Select one option)

- ○ 1 - Completely Disagree
- ○ 2 – Disagree
- ○ 3 – Neutral /Do not have an opinion
- ○ 4 – Agree
- ○ 5- Completely Agree

* **17.** **12. In your opinion does on-farm breeding cause important ENVIRONMENTAL issues e.g. huge water consumption and greenhouse gas emissions?** ***Please tick the one option that most applies to you***

(Select one option)

- ○ 1 - Completely Disagree
- ○ 2 – Disagree
- ○ 3 – Neutral /Do not have an opinion
- ○ 4 – Agree
- ○ 5- Completely Agree

* **18.** **13. In your opinion can the potential problems of on-farm breeding be dealt with by reducing our meat consumption?** ***Please tick the one option that most applies to you***

(Select one option)

- ○ 1 - Completely Disagree
- ○ 2 – Disagree
- ○ 3 – Neutral /Do not have an opinion
- ○ 4 – Agree
- ○ 5- Completely Agree

* **19.** **14. And do you believe that if people ate Artificial Meat instead of conventional/ normal meat, it would improve the state of animals and reduce animal suffering?** ***Please tick the one option that most applies to you***

(Select one option)

- ○ 1 - Completely Disagree
- ○ 2 – Disagree
- ○ 3 – Neutral /Do not have an opinion
- ○ 4 – Agree
- ○ 5 - Completely Agree

* **20.** **15. Using the following rating scale, do you think that Artifical meat could negatively impact live stock farming and the meat industry (e.g. by reducing the number of jobs available)?** ***Please tick the one option that most applies to you***

(Select one option)

- ○ 1 – Completely Disagree
- ○ 2 – Disagree
- ○ 3 – Neutral /Do not have an opinion
- ○ 4 – Agree
- ○ 5- Completely Agree

* **21. 16. Do you think that Artificial meat would have a negative impact on rural life?** ***Please tick the option that most applies to you***

(Select one option)

- ◯ 1 - Completely Disagree
- ◯ 2 – Disagree
- ◯ 3 – Neutral /Do not have an opinion
- ◯ 4 – Agree
- ◯ 5- Completely Agree

* **22.** **17. To what extent do you believe that Artificial Meat would be healthier& have higher nutritional value than normal meat?** ***Please tick the one option that most applies to you*** **(Select one option)**

- ○ 1 - Completely Disagree
- ○ 2 – Disagree
- ○ 3 – Neutral /Do not have an opinion
- ○ 4 – Agree
- ○ 5- Completely Agree

* **23.** **18. In your opinion do you believe that Artificial Meat is more tasty compared to normal meat.** ***Please tick the one option that most applies to you***

(Select one option)

- ○ 1 - Completely Disagree
- ○ 2 – Disagree
- ○ 3 – Neutral /Do not have an opinion
- ○ 4 – Agree
- ○ 5 - Completely Agree

POTENTIAL INTERESTS

* **24.** **19. Would you accept Artificial Meat as a viable alternative to normal meat in the future (Just like other meat substitute like Soy proteins)?** ***Please tick the one option that most applies to you***

(Select one option)

- ○ Yes, I already eat meat substitute or meat alternatives
- ○ Yes, but I do not eat meat substiute or meat alternatives
- ○ No, but I eat meat and meat alternatives
- ○ No, I do not eat meat substitutes and/or meat alternatives

* **25.** **20. Which of the following reasons would be most likely to persuade you to try artificial meat?** ***Please tick ALL the options that you agree with***

- ☐ As a solution to feed the ever growing human population
- ☐ It has more attractive pricing than conventional meat
- ☐ Ethics – it improves the well being of animals and reduces animal slaughtering
- ☐ Less risk of Zoonosis (diseases that can be transmitted from animals to people e.g. Foot & mouth disease)
- ☐ Attractiveness of high-tech technologies
- ☐ The artificial meat product is a clean product
- ☐ Artificial meat is an Eco-friendly product
- ☐ Curiosity
- ☐ Other reasons
- ☐ I am not willing to try artificial meat

NOTE : Answer the below question only if answer to Q#25 is Other reasons

* **26.** **Other reasons (Please specify)**

NOTE : Answer the below question only if answer to Q#25 is I am not willing to try artificial meat

* **27.** **21. And which of the following reasons would be the most likely reasons that you are not willing to try artificial meat?** ***Please tick ALL the options that you agree with***

- ☐ It is unnatural
- ☐ It is less tasty/appealing
- ☐ I am worried about its safety
- ☐ It is more expensive than normal meat
- ☐ I am reluctant (feel disgusted/nervous)
- ☐ Negative impact on local farmers & their jobs
- ☐ Negative impact on rural life
- ☐ I do not trust laboratories and articial meat start-up companies
- ☐ It has significant environmental footprint
- ☐ Negative impact on land reserves and rural life e.g biodiversity, tourism, land scape maintenance
- ☐ Other (Please specify) _______________

* **28.** **22. Which of the following statements would you associate with artificial meat? We are just looking for your opinion, even though you may have had little experience with Artificial meat.** ***Please tick ALL the options that you agree with***

- ☐ Adequate nutrition
- ☐ Tasty /tastes similar to real/normal meat
- ☐ Safety
- ☐ Less as a solution to feed the ever growing human population
- ☐ It is less expensive or has better pricing than conventional meat
- ☐ It has less environmental footprint
- ☐ Leads to the reduction of farming
- ☐ Requires no farming
- ☐ There is no animal pain, or suffering
- ☐ Other (Please specify)
- ☐ Nothing

NOTE : Answer the below question only if answer to Q#28 is Other (Please specify)

* **29.** **Other (Please specify)**

* **30.** **23. Now that you have learnt a little bit more about artificial meat, what do you think about it?** ***Please tick the ONE statement that you agree with the most*** **(Select one option)**

- ○ It is promising and/or acceptable
- ○ It is fun and/or intriguing
- ○ It is absurd and/or disgusting

* **31.** **24. In your opinion would you say that you have emotional resistance to trying out artificial meat (e.g. disgust or nervous)?** ***Please tick the one option that you agree with*** **(Select one option)**

- ○ 1 - Completely Disagree
- ○ 2 – Disagree
- ○ 3 – Neutral /Do not have an opinion
- ○ 4 – Agree
- ○ 5 - Completely Agree

* **32.** **25. Would you be willing to try out artificial meat?** ***Please tick the one option that you most agree with***
(Select one option)

- ○ 1 – Will definitely try
- ○ 2 – Will probably try
- ○ 3 – Unsure or Undecided
- ○ 4 – Will probably not try
- ○ 5 - Will definitely not try

* **33.** **26. Which of the following cases are you most likely to eat artificial meat regularly?** ***Please tick all options that you agree with***

- [ ] At the restaurant
- [ ] At home
- [ ] In prepackaged ready-to-eat meals (e.g. Spaghetti and mincemeat)
- [ ] Other
- [ ] I do not want to eat artificial meat regularly

* **34.** **27. Compared to the price of normal/conventional meat how much are you willing to pay for artificial meat?** ***Please tick the one option that you most agree with*** **(Select one option)**

- ◯ Much less than what I would pay for conventional meat, even nothing at all
- ◯ Less than for coventional meat
- ◯ Same price as for conventional meat
- ◯ More than what I would pay for conventional meat
- ◯ Much more than what I would pay for conventional meat

* **35.** **28. Artificial meat is already available in some countries, what timeline do you believe that artificial meat will be widely accepted?** ***Please tick the one option that you most agree with***
(Select one option)

- ○ In the short term – 1 to 5 years
- ○ In the medium term – 6 to 15 years
- ○ In the long term – more than 15 years
- ○ Never

*** 36. 29. Below is a list of names which are most commonly used to refer to artificial meat. Given what you know of artificial meat, which of the following names do you believe is most suitable for artificial meat?**
Please tick ALL the options that you agree with

- ☐ Artificial Meat
- ☐ In-vitro Meat
- ☐ Clean Meat
- ☐ Cultured Meat
- ☐ Cellular Meat
- ☐ Lab meat
- ☐ Synthetic Meat
- ☐ Animal-free meat
- ☐ Slaughter-free meat

* **37. 30. The Private research model is when scientific research is supported by private money for instance from rich people to private companies. To which extent do you agree that the private research model is relevant for developing research on artificial meat?** ***Please tick the ONE option that you most agree with*** **(Select one option)**

- ○ 1 - Completely Disagree
- ○ 2 – Disagree
- ○ 3 – Unsure /Do not have an opinion
- ○ 4 – Agree
- ○ 5 - Completely Agree

* **38.** **31. The Public research model is when scientific research is supported by public grants given to research organizations by governments or public agencies. To which extent do you believe that scientific public research must invest (time and money) to develop this biotechnology)?** ***Please tick the ONE option that you agree with*** **(Select one option)**

- ○ 1 - Completely Disagree
- ○ 2 – Disagree
- ○ 3 – Unsure /Do not have an opinion
- ○ 4 – Agree
- ○ 5 - Completely Agree

* **39.** **32. If this product is commercialized one day, do you think it should be labelled as ''meat''?** ***Please tick the ONE option that you most agree with*** **(Select one option)**

- ○ Yes
- ○ No

* **40.** **33. Do you agree that the information you provided today will be saved? All information will remain anonymous and confidential and cannot be linked to you.** ***Please tick the ONE option that you agree with*** **(Select one option)**

- ○ Yes
- ○ No

**NOTE :** IF ANSWER TO **Q40** is<br>**Yes** Stop, you have finished the survey<br>**No** Stop, you have finished the survey

La viande artificielle est également connue sous le nom de viande in vitro, de viande de culture, de viande de laboratoire, de viande propre ou de viande synthétique. C'est de la viande de laboratoire, où la « viande » est cultivée à l'extérieur du corps de l'animal, à l'aide de cellules souches des muscles de l'animal (voir l'image ci-dessous). Cela nous permet donc d'obtenir de la viande sans avoir à abattre des vaches ou d'autres animaux. Il y a beaucoup d'intérêt pour la viande artificielle car elle peut aider à résoudre la faim dans le monde en fournissant de la nourriture pour la population humaine croissante. Il contribuera également à résoudre les problèmes environnementaux croissants (p. ex., le réchauffement climatique) et d'autres problèmes éthiques, car il améliore la vie des animaux en raison de la réduction des abattages. Pour ces raisons, la recherche scientifique est consacrée au développement de la viande artificielle comme source viable de viande pour l'avenir.

étude est menée par des chercheurs français de l'INRAE (Theix, 63), de l'ISARA (Lyon, 69) et de Bordeaux Sciences-Agro (Bordeaux, 33). Le but de cette enquête est de comprendre comment les gens ordinaires comme vous se sentent au sujet de cette nouvelle biotechnologie et de savoir quelles sont vos préférences et vos préoccupations. C'est un sujet intéressant, et il ne devrait pas prendre plus de 5 minutes pour vous de compléter. Si vous avez des questions, vous pouvez écrire à l'équipe de recherche à « futurecellmeat@gmail.com ». Merci de votre participation!

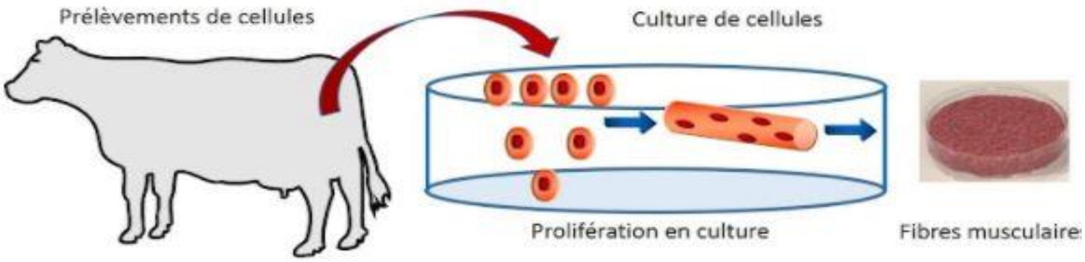

.

* **41.** **7. Comment êtes-vous impliqué dans le secteur de la viande?** ***Veuillez cocher la seule option qui s'applique le plus à vous.*** **(Select one option)**

- ○ Je suis un scientifique, qui travaille dans le secteur de la viande
- ○ Je suis un scientifique qui travaille à l'extérieur du secteur de la viande.
- ○ Je ne suis pas un scientifique et je travaille dans le secteur de la viande.
- ○ Je ne suis pas un scientifique et je ne travaille pas dans le secteur de la viande.

* **42.** **8. À quelle fréquence mangez-vous de la viande?** ***Veuillez cocher la seule option qui s'applique le plus à vous.*** **(Select one option)**

- ◯ Je ne mange jamais de viande – Je suis Végétarien ou végétalien
- ◯ Je mange rarement de la viande – une fois par semaine ou moins
- ◯ Je mange régulièrement ensemble – plusieurs fois par semaine
- ◯ Je mange de la viande tous les jours - ou à chaque repas

* **43.** **9. Avant aujourd'hui, aviez-vous déjà entendu parler de la viande artificielle?** ***Veuillez cocher la seule option qui s'applique le plus à vous.***

(Select one option)

- ○ Oui
- ○ Non

* **44.** **10. Parmi les facteurs suivants, lesquels sont importants pour vous lorsque vous allez acheter de la viande?**
Veuillez cocher TOUTES les options qui s'appliquent le plus à vous.

- ☐ Éthique de la façon dont la viande a été produite, p. ex., les animaux ont-ils pu se déplacer librement?
- ☐ Impact environnemental de la nourriture/viande pendant sa production
- ☐ Prix
- ☐ Qualité de la viande (goût, jus, tendresse) …
- ☐ Aspect de la viande (p.ex. sa couleur, sa fraîcheur)
- ☐ Consommation d'énergie de l'animal pendant la production (Calories utilisées)
- ☐ Valeur nutritive de la viande (p. ex., nombre de protéines, vitamines)
- ☐ Origines et traçabilité de la viande
- ☐ Sécurité
- ☐ Information sur les étiquettes nutritionnelles, les étiquettes d'aliments ou les marques
- ☐ Autre

DÉFIS SOCIÉTAUX

Nous allons maintenant examiner vos perceptions de la façon dont la viande est produite, en nous concentrant sur la reproduction des animaux à la ferme. L'élevage d'animaux de ferme est l'accouplement sélectif d'animaux pour augmenter la possibilité d'obtenir certaines caractéristiques souhaitées chez la progéniture de l'animal. Pour chaque réponse, l'échelle de réponse va de 1 à 5, 1 signifiant que vous n'êtes pas du tout d'accord, et 5 que vous êtes tout à fait d'accord

* **45.** **11. Croyez-vous que l'élevage à la ferme peut causer d'importants problèmes ÉTHIQUES, (p. ex, les animaux qui souffrent, les animaux qui sont abattus)?** ***Veuillez cocher la seule option qui s'applique le plus à vous***

(Select one option)

- ◯ 1 - Complètement en désaccord
- ◯ 2 – En désaccord
- ◯ 3 – Neutre / Pas d'opinion
- ◯ 4 – D'accord
- ◯ 5- Entièrement d'accord

* **46.** **12. À votre avis, l’élevage à la ferme cause-t-il des problèmes ENVIRONNEMENTAUX importants p. ex., énorme consommation d’eau et émissions de gaz à effet de serre?** ***Veuillez cocher la seule option qui s’applique le plus à vous***

(Select one option)

- ○ 1 - Complètement en désaccord
- ○ 2 – En désaccord
- ○ 3 – Neutre / Pas d’opinion
- ○ 4 – D’accord
- ○ 5- Entièrement d’accord

* **47.** **13. À votre avis, peut-on régler les problèmes potentiels de la sélection à la ferme en réduisant notre consommation de viande?** ***Veuillez cocher la seule option qui s'applique le plus à vous.***

(Select one option)

- ○ 1 - Complètement en désaccord
- ○ 2 – En désaccord
- ○ 3 – Neutre / Pas d'opinion
- ○ 4 – D'accord
- ○ 5- Entièrement d'accord

* **48.** **14. Croyez-vous que si les gens mangeaient de la viande artificielle au lieu de la viande conventionnelle/normale, cela améliorerait la condition des animaux et réduirait leur souffrance ?** ***Veuillez cocher l'option qui vous concerne le plus.***

(Select one option)

- ○ 1 - Complètement en désaccord
- ○ 2 – En désaccord
- ○ 3 – Neutre / Pas d’opinion
- ○ 4 – D’accord
- ○ 5 - Entièrement d’accord

* **49.** **15. En utilisant l'échelle de cotation suivante, pensez-vous que la viande artificielle pourrait avoir un impact négatif sur l'élevage vivant et l'industrie de la viande (p. ex., en réduisant le nombre d'emplois disponibles)?** ***Veuillez cocher la seule option qui s'applique le plus à vous.***

(Select one option)

- ○ 1 – Complètement en désaccord
- ○ 2 – En désaccord
- ○ 3 – Neutre / Pas d'opinion
- ○ 4 – D'accord
- ○ 5- Entièrement d'accord

* **50.** **16. Pensez-vous que la viande artificielle aurait un impact négatif sur la vie rurale?** ***Veuillez cocher l'option qui s'applique le plus à vous.***

(Select one option)

- ◯ 1 - Complètement en désaccord
- ◯ 2 – En désaccord
- ◯ 3 – Neutre / Pas d'opinion
- ◯ 4 – D'accord
- ◯ 5- Entièrement d'accord

* **51.** **17. Dans quelle mesure croyez-vous que la viande artificielle serait plus saine et aurait une valeur nutritive plus élevée que la viande normale?** ***Veuillez cocher la seule option qui s'applique le plus à vous.***

(Select one option)

- ○ 1 - Complètement en désaccord
- ○ 2 – En désaccord
- ○ 3 – Neutre / Pas d'opinion
- ○ 4 – D'accord
- ○ 5- Entièrement d'accord

* **52.** **18. Et enfin, à votre avis, croyez-vous que la viande artificielle est plus savoureuse par rapport à la viande normale?** ***Veuillez cocher la seule option qui s'applique le plus à vous.***

(Select one option)

- ○ 1 - Complètement en désaccord
- ○ 2 – En désaccord
- ○ 3 – Neutre / Pas d'opinion
- ○ 4 – D'accord
- ○ 5 - Entièrement d'accord

INTÉRÊTS POTENTIELS

* **53.** **19. Accepteriez-vous la viande artificielle comme alternative viable à la viande normale à l'avenir? (Tout comme d'autres substituts de viande comme les protéines de soya)?** ***Veuillez cocher la seule option qui s'applique le plus à vous.***

(Select one option)

- ◯ Oui, je mange déjà des substituts de viande ou des substituts de viande
- ◯ Oui, mais je ne mange pas de substituts de viande ou de substituts de viande
- ◯ Non, mais je mange de la viande et/ou des substituts de viande
- ◯ Non, je ne mange pas de substituts de viande et/ou de substituts de viande

* **54.** **20. Laquelle des raisons suivantes serait le plus susceptible de vous persuader d'essayer la viande artificielle?** ***Veuillez cocher TOUTES les options avec lesquelles vous êtes d'accord***

- ☐ Comme une solution pour nourrir la population humaine toujours croissante
- ☐ Il a des prix plus attrayants que la viande conventionnelle
- ☐ Éthique – améliore le bien-être des animaux et réduit l'abattage des animaux
- ☐ Risque moindre de zoonose (maladie qui peut être transmise des animaux aux humains, p. ex., fièvre aphteuse)
- ☐ Attractivité des technologies de pointe
- ☐ Le produit de viande artificielle est un produit propre
- ☐ La viande artificielle est un produit écologique
- ☐ Curiosité
- ☐ Autres raisons
- ☐ Je ne suis pas prêt à essayer de la viande artificielle?

NOTE : Answer the below question only if answer to Q#54 is Autres raisons

* **55.** **Autres raisons (veuillez préciser)**

NOTE : Answer the below question only if answer to Q#54 is Je ne suis pas prêt à essayer de la viande artificielle?

* **56.** **21. Et laquelle des raisons suivantes serait la plus probable que vous n'êtes pas prêt à essayer la viande artificielle?** ***Veuillez cocher TOUTES les options avec lesquelles vous êtes d'accord***

- ☐ C'est contre nature
- ☐ Il est moins savoureux/attrayant
- ☐ Je m'inquiète pour sa sécurité
- ☐ Il est plus cher que la viande normale
- ☐ Je suis réticent (dégoûté/nerveux)
- ☐ Elle a un impact négatif sur les emplois des agriculteurs locaux
- ☐ Impact négatif sur les agriculteurs locaux et leurs emplois
- ☐ Impact négatif sur la vie rurale
- ☐ Je ne fais pas confiance aux laboratoires et aux jeunes entreprises de viande artificielle
- ☐ Il a une empreinte environnementale importante
- ☐ Impact négatif sur les réserves foncières et la vie rurale, par exemple la biodiversité, le tourisme, l'entretien du paysage.
- ☐ Autre (veuillez préciser) _______________

* **57.** **22. Lequel des énoncés suivants associez-vous à la viande artificielle? Nous sommes à la recherche de votre avis, même si vous avez peut-être eu peu d'expérience avec la viande artificielle?** ***Veuillez cocher TOUTES les options avec lesquelles vous êtes d'accord***

- ☐ Nutrition adéquate
- ☐ Savoureux/goût semblable à la viande réelle/normale
- ☐ Sécurité
- ☐ Moins comme solution pour nourrir la population humaine toujours croissante
- ☐ Il est moins cher ou a un meilleur prix que la viande conventionnelle
- ☐ Il a moins d'empreinte environnementale
- ☐ Conduit à la réduction des famings
- ☐ Ne nécessite pas d'encadrement
- ☐ Il n'y a ni douleur animale, ni souffrance
- ☐ Autre (préciser)
- ☐ Rien

NOTE : Answer the below question only if answer to Q#57 is Autre (préciser)

* **58.** **Autre (préciser)**

* **59.** **23. Maintenant que vous avez appris un peu plus sur la viande artificielle, que pensez-vous à ce sujet?**
Veuillez cocher l'énoncé ONE que vous approuvez le plus.
(Select one option)

- ○ C'est prometteur et/ou acceptable
- ○ C'est amusant et/ou intrigant
- ○ C'est absurde et/ou dégoûtant

* **60.** **24. À votre avis, diriez-vous que vous avez une résistance émotionnelle à essayer de la viande artificielle (p. ex., dégoût ou nerveux)?** ***Veuillez cocher la seule option que vous approuvez.*** **(Select one option)**

- ◯ 1 - Complètement en désaccord
- ◯ 2 – En désaccord
- ◯ 3 – Neutre / Pas d’opinion
- ◯ 4 – D’accord
- ◯ 5 - Entièrement d’accord

*** 61.** **25. Seriez-vous prêt à essayer de la viande artificielle?** ***Veuillez cocher la seule option que vous approuvez le plus*** **(Select one option)**

- ○ 1 – Va certainement essayer
- ○ 2 – Essaiera probablement
- ○ 3 – Incertain ou indécis
- ○ 4 – Ne tentera probablement pas
- ○ 5 - Ne tentera certainement pas

* **62.** **26. Lequel des cas suivants êtes-vous le plus susceptible de manger de la viande artificielle régulièrement?**
Veuillez cocher toutes les options que vous acceptez.

- ☐ Au restaurant
- ☐ À la maison
- ☐ Dans les repas prêts-à-manger préemballés (p. ex. spaghetti et viande hachée)
- ☐ Autre
- ☐ Je ne veux pas manger de viande artificielle régulièrement

* **63.** **27. Par rapport au prix de la viande normale/conventionnelle, combien êtes-vous prêt à payer pour la viande artificielle?** ***Veuillez cocher la seule option que vous approuvez le plus*** **(Select one option)**

- ○ Beaucoup moins que ce que je paierais pour la viande conventionnelle, même rien du tout
- ○ Moins que pour les viandes conventionnelles
- ○ Même prix que pour la viande conventionnelle
- ○ Plus que ce que je paierais pour la viande conventionnelle
- ○ Beaucoup plus que ce que je paierais pour la viande conventionnelle

* **64.** **28. La viande artificielle est déjà disponible dans certains pays; quel délai croyez-vous que la viande artificielle sera largement acceptée?** ***Veuillez cocher la seule option que vous approuvez le plus*** **(Select one option)**

- À court terme – 1 à 5 ans
- À moyen terme – 6 à 15 ans
- À long terme – plus de 15 ans
- Jamais

* **65.** **29. Voici une liste de noms qui sont les plus couramment utilisés pour se référer à la viande artificielle. Compte tenu de ce que vous savez de la viande artificielle, lequel des noms suivants croyez-vous est le plus approprié pour la viande artificielle?**
Veuillez cocher TOUTES les options avec lesquelles vous êtes d'accord

- ☐ Viande artificielle
- ☐ Viande in vitro
- ☐ Viande propre
- ☐ Viande de culture
- ☐ Viande cellulaire
- ☐ Viande de laboratoire
- ☐ Viande synthétique
- ☐ Viande exempte d'animaux
- ☐ Viande non abattue

* **66.** **30. Le modèle de la recherche privée est quand la recherche scientifique est soutenue par l'argent privé par exemple des personnes riches aux entreprises privées. Dans quelle mesure êtes-vous d'accord pour dire que le modèle de recherche privé est pertinent pour le développement de la recherche sur la viande artificielle?** ***Veuillez cocher la SEULE option que vous approuvez le plus.*** **(Select one option)**

- ◯ 1 - Complètement en désaccord
- ◯ 2 – En désaccord
- ◯ 3 – Incertain/Pas d'opinion
- ◯ 4 – D'accord
- ◯ 5 - Entièrement d'accord

* **67. 31. Le modèle de la recherche publique s'applique lorsque la recherche scientifique est appuyée par des subventions publiques accordées à des organismes de recherche par des gouvernements ou des organismes publics. Dans quelle mesure croyez-vous que la recherche scientifique publique doit investir (temps et argent) pour développer cette biotechnologie?** ***Veuillez cocher la SEULE option que vous approuvez.***
(Select one option)

- ○ 1 - Complètement en désaccord
- ○ 2 – En désaccord
- ○ 3 – Incertain/Pas d'opinion
- ○ 4 – D'accord
- ○ 5 - Entièrement d'accord

* **68.** **32. Si ce produit est commercialisé un jour, pensez-vous qu'il devrait être étiqueté comme étant "de la viande"?** ***Veuillez cocher la SEULE option que vous approuvez le plus.*** **(Select one option)**

- ○ Oui
- ○ Non

* **69.** **33. Convenez-vous que les renseignements que vous avez fournis aujourd'hui seront sauvegardés? Toutes les informations demeureront anonymes et confidentielles et ne peuvent pas être liées à vous.** ***Veuillez cocher la SEULE option que vous approuvez.*** **(Select one option)**

- ◯ Oui
- ◯ Non
