# Supplementary material for: Consumer perception of “artificial meat” in the educated young and urban population of Africa
Source: Front Nutr. 2023 Apr 14;10:1127655. doi: 10.3389/fnut.2023.1127655 (PMC10140314; doi:10.3389/fnut.2023.1127655)
Supplement: Supplementary file 2 [file Table_1.DOCX]

**Table 1: Distribution of responses with respect to socio-demographics**

|  |  | **Survey 1** | | **Survey 2** | | **Survey 3** | |
| --- | --- | --- | --- | --- | --- | --- | --- |
| **Question** | **Response option** | **Number of responses** | **Percentage**  **(%)*** | **Number of responses** | **Percentage**  **(%)*** | **Number of responses** | **Percentage**  **(%)*** |
| Gender | Female  Male | 2725  2760 | 49.7  50.3 | 2602  2926 | 47.1  52.9 | 464  647 | 41.8  58.2 |
| Age | 18-30 years  31-50 years  >51 years | 1583  3745  157 | 28.9  68.3  2.8 | 1763  3593  172 | 31.9  65.0  3.1 | 517  544  50 | 46.5  49.0  4.5 |
| Education | Primary School  High school  Undergraduate  Technical Training  Graduate | 216  412  149  1490  3218 | 3.9  7.5  2.7  27.1  58.7 | 205  410  168  1615  3130 | 3.7  7.4  3.1  29.2  56.6 | 27  52  25  324  683 | 2.4  4.7  2.25  29.2  61.5 |
| Monthly income | Under USD1,500  More than USD1,500 | 3208  2277 | 58.5  41.5 | 3179  2349 | 57.5  42.5 | 484  627 | 43.6  56.4 |
| Total |  | **5485** | **100** | **5528** | **100** | **1111** | **100** |

*Percentage of people who answered the questionnaire

**Table 2: Respondents' perspective with respect to societal challenges in both surveys**

| **Question** |  | **Response (1: completely disagree – 5: completely agree)** | | | | |
| --- | --- | --- | --- | --- | --- | --- |
|  |  | **1** | **2** | **3** | **4** | **5** |
| In your opinion does on-farm breeding cause important ENVIRONMENTAL issues e.g.  huge water consumption and greenhouse gas emissions? | Survey 1 | 794  (14.5%) | 1579  (28.8%) | 1727  (31.5%) | 1138  (20.7%) | 246  (4.5%) |
|  | Survey 2 | 221  (19.9%) | 334  (30.1%) | 370  (33.3%) | 159  (14.3%) | 27  (2.4%) |
| Do you believe that on-farm breeding can cause important ETHICAL problems, e.g. animal suffering, animal slaughter)? | Survey 1 | 859  (15.7%) | 1813  (33.1%) | 1527  (27.8%) | 1056  (19.3%) | 229  (4.2%) |
|  | Survey 2 | 301  (27.1%) | 267  (25%) | 376  (33.8%) | 147  (13.2%) | 20  (1.8%) |
| In your opinion, can the potential problems of on-farm breeding be dealt with by reducing our meat consumption? | Survey 1 | 735  (13.4%) | 1420  (25.9%) | 1717  (31.3%) | 1314  (24%) | 298  (5.4%) |
|  | Survey 2 | 243  (21.9%) | 293  (26.4%) | 374  (33.7%) | 179  (16.1%) | 22  (2%) |
| Do you believe that if people ate Artificial meat instead of conventional meat, it would improve the welfare of animals and reduce animal suffering? | Survey 1 | 656  (12%) | 1246  (22.7%) | 1790  (32.6%) | 1437  (26.2%) | 355  (6.5%) |
|  | Survey 2 | 252  (22.7%) | 276  (24.8%) | 383  (34.5%) | 180  (16.2%) | 20  (1.8%) |
| Using the following rating scale, do you think that Artificial meat could negatively impact livestock farming and the meat industry (e.g. by reducing the number of jobs available? | Survey 1 | 547  (10%) | 1055  (19.2%) | 1599  (29.1%) | 1764  (32.2%) | 519  (9.5%) |
|  | Survey 2 | 239  (21.5%) | 270  (24.3%) | 343  (30.9%) | 205  (18.4%) | 54  (4.9%) |
| Do you think that Artificial meat would have a negative impact on rural life? | Survey 1 | 503  (9.2%) | 1098  (20%) | 1570  (28.6%) | 1713  (31.2%) | 600  (10.9%) |
|  | Survey 2 | 201  (18.1%) | 278  (25%) | 332  (29.9%) | 231  (20.8%) | 69  (6.2%) |
| To what extent do you believe that Artificial meat would be healthier & have higher nutritional value than normal meat? | Survey 1 | 724  (13.2%) | 1291  (23.5%) | 1913  (34.9%) | 1185  (21.6%) | 371  (6.8%) |
|  | Survey 2 | 292  (26.3%) | 264  (23.8%) | 397  (35.7%) | 132  (11.8%) | 26  (2.3%) |
| In your opinion do you believe that Artificial meat is tastier compared to normal meat. | Survey 1 | 1032  (18.8%) | 1171  (21.3%) | 1898  (34.6%) | 1095  (20%) | 288  (5.2%) |
|  | Survey 2 | 284  (25.6%) | 297  (26.7%) | 399  (35.9%) | 118  (10.6%) | 13  (1.2%) |
| In your opinion would you say that you have emotional resistance to trying out Artificial meat (e.g. disgust or nervousness)? | Survey 1 | 548  (9.9%) | 1516  (27.4%) | 2197  (39.7%) | 853  (15.4%) | 414  (7.5%) |
|  | Survey 2 | 294  (26.5%) | 288  (25.9%) | 371  (33.4%) | 112  (10.1%) | 46  (4.1%) |

**Table 3: Potential interest in “artificial meat” in both surveys**

|  |  | Survey 1 and 2 (n=11013) | | Survey 3 (n=1111) | |
| --- | --- | --- | --- | --- | --- |
| Question | Response options | Number of responses | Percentages (%) | Number of responses | Percentages (%) |
| Which of the following would you say are important considerations for you when you go to shop for meat?  Which of the following would you say are important considerations for you when you go to shop for meat?  (multiple choice question) | Ethics of how the meat was produced, e.g. were the animals allowed to roam freely | 923 | 5.5 | 491 | 12.5 |
|  | Environmental impact of the food/meat during its production | 1572 | 9.4 | 501 | 12.7 |
|  | Price | 3150 | 18.8 | 792 | 20.1 |
|  | Quality of the meat (taste, juiciness, tenderness) … | 2884 | 17.2 | 624 | 15.9 |
|  | Appearance of the meat (e.g. its color, freshness) | 2220 | 13.3 | 483 | 12.3 |
| Would you accept Artificial Meat as a viable alternative to normal meat in the future (Just like other meat substitutes like Soy proteins)? | Yes, I already eat meat substitutes or meat alternatives | 837 | 15.3 | 278 | 25.1 |
|  | Yes, but I do not eat meat substitutes or meat alternatives | 1579 | 28.8 | 371 | 33.4 |
|  | No, but I eat meat and/or meat alternatives | 1804 | 32.9 | 286 | 25.7 |
|  | No, I do not eat meat substitutes and/or meat alternatives | 1265 | 23.1 | 176 | 15.8 |
| Which of the following reasons would be most likely to persuade you to try Artificial meat?* | As a solution to feed the ever-growing human population | 1647 | 12.2 | 445 | 14.4 |
|  | It has more attractive pricing than conventional meat | 1646 | 12.2 | 509 | 16.4 |
|  | Ethics – it improves the wellbeing of animals and reduces animal slaughter | 1861 | 13.8 | 535 | 17.3 |
|  | Less risk of Zoonosis (disease that can be transmitted from animals to people e.g. Foot & mouth  disease) | 1630 | 12.1 | 462 | 14.9 |
|  | Attractiveness of high-tech technologies | 1371 | 10.1 | 330 | 10.6 |
|  | Curiosity | 1584 | 11.7 | 260 | 8.4 |
| And which of the following would be the most likely reasons why you would not be willing to try Artificial meat?* | It is unnatural | 1756 | 12.7 | 47 | 20.0 |
|  | It is less tasty/appealing | 1587 | 11.4 | 15 | 6.4 |
|  | I am worried about its safety | 1929 | 13.9 | 29 | 12.3 |
|  | It is more expensive than normal meat | 1667 | 12.0 | 15 | 6.4 |
|  | I am reluctant (feel disgusted/nervous) | 1584 | 11.4 | 22 | 9.4 |
|  | It has a negative impact on local farmers | 1546 | 11.2 | 22 | 9.4 |
|  | Negative impact on local farmers & their jobs | 1426 | 10.3 | 28 | 11.9 |
|  | I do not trust laboratories and artificial meat start-up companies | 966 | 7.0 | 37 | 15.7 |
| Which of the following statements would you associate with Artificial meat?* | Adequate nutrition | 1241 | 9.0 | 468 | 13.5 |
|  | Tasty /tastes similar to real/normal meat | 1527 | 9.2 | 497 | 14.3 |
|  | Safety | 1923 | 14.0 | 626 | 18.1 |
|  | Less as a solution to feed the ever-growing human population | 1548 | 11.3 | 452 | 13.0 |
|  | It is less expensive or has better pricing than conventional meat | 1354 | 9.9 | 406 | 11.7 |
|  | It has a smaller environmental footprint | 1267 | 9.2 | 327 | 9.4 |
|  | Leads to the reduction of farming | 1161 | 8.5 | 264 | 7.6 |
|  | Leads to no farming | 1038 | 7.6 | 218 | 6.3 |
| In which of the following cases would you be most likely to eat Artificial meat regularly? | At the restaurant | 1323 | 23.9 | 462 | 29.3 |
|  | At home | 1431 | 25.9 | 474 | 30.0 |
|  | In prepackaged ready-to-eat meals (e.g. lasagna…) | 1101 | 19.9 | 372 | 23.6 |
|  | I do not want to eat artificial meat regularly | 821 | 14.8 | 141 | 8.9 |
|  | Other | 852 | 15.4 | 129 | 8.2 |
| Now that you have learnt a little bit more about Artificial meat, what do you think about it? | It is promising and / or acceptable | 1257 | 22.7 | 437 | 39.3 |
|  | It is fun and/or intriguing | 3262 | 59 | 497 | 44.7 |
|  | It is absurd and/or disgusting | 1009 | 18.2 | 177 | 15.9 |
| Artificial meat is already available in some countries, when do you think artificial meat will be widely accepted? | In the short term – 1 to 5 years | 1208 | 21.8 | 408 | 36,7 |
|  | In the medium term – 6 to 15 years | 2381 | 43.0 | 345 | 31.0 |
|  | In the long term – more than 16 years | 1124 | 20.3 | 261 | 23.5 |
|  | Never | 815 | 14.7 | 97 | 8.7 |

*Only major answers are indicated
